# The genetic basis of DOORS syndrome: an exome-sequencing study

**DOI:** 10.1016/S1474-4422(13)70265-5

**Published:** 2014-01

**Authors:** Philippe M Campeau, Dalia Kasperaviciute, James T Lu, Lindsay C Burrage, Choel Kim, Mutsuki Hori, Berkley R Powell, Fiona Stewart, Têmis Maria Félix, Jenneke van den Ende, Marzena Wisniewska, Hülya Kayserili, Patrick Rump, Sheela Nampoothiri, Salim Aftimos, Antje Mey, Lal D V Nair, Michael L Begleiter, Isabelle De Bie, Girish Meenakshi, Mitzi L Murray, Gabriela M Repetto, Mahin Golabi, Edward Blair, Alison Male, Fabienne Giuliano, Ariana Kariminejad, William G Newman, Sanjeev S Bhaskar, Jonathan E Dickerson, Bronwyn Kerr, Siddharth Banka, Jacques C Giltay, Dagmar Wieczorek, Anna Tostevin, Joanna Wiszniewska, Sau Wai Cheung, Raoul C Hennekam, Richard A Gibbs, Brendan H Lee, Sanjay M Sisodiya

**Affiliations:** a Department of Molecular and Human Genetics, Baylor College of Medicine, Houston, TX, USA; b Department of Clinical and Experimental Epilepsy, UCL Institute of Neurology, London, UK; c Human Genome Sequencing Center, Baylor College of Medicine, Houston, TX, USA; d Department of Structural and Computational Biology and Molecular Biophysics, Baylor College of Medicine, Houston, TX, USA; e Department of Pharmacology, Baylor College of Medicine, Houston, TX, USA; f Department of Pediatrics, Toyohashi Municipal Hospital, Toyohashi, Aichi, Japan; g Children's Hospital Central California, Madera, California, USA; h Genetics Service, Belfast City Hospital, Belfast, Ireland; i Medical Genetics Service, Clinical Hospital of Porto Alegre, Porto Alegre, Brazil; j Department of Medical Genetics, University Hospital Antwerp, 2650 Antwerp, Belgium; k Department of Medical Genetics, Poznañ University of Medical Sciences, Poznañ, Poland; l Medical Genetics Department, Istanbul Medical Faculty, Istanbul University, Turkey; m Department of Genetics, University of Groningen, Groningen, Netherlands; n Department of Pediatric Genetics, Amrita Institute of Medical Sciences and Research Centre, Kerala, India; o Genetic Health Service New Zealand—Northern Hub, Auckland City Hospital, Auckland, New Zealand; p Pediatric Neurology, Braunschweig Hospital, Braunschweig, Germany; q Department of Pediatrics, Saveetha Medical College and Hospital, Saveetha University, Chennai, Tamil Nadu, 600077, India; r Division of Genetics, Children's Mercy Hospitals and Clinics and the University of Missouri-Kansas City School of Medicine, Kansas City, MO, USA; s Department of Medical Genetics, Montreal Children's Hospital, McGill University Health Center, Quebec, Canada; t Department of Pediatrics, NKP Salve Institute of Medical Sciences and Lata Mangeshkar Hospital, Maharashtra, India; u University of Washington Medical Center, Seattle, WA, USA; v Center for Human Genetics, Facultad de Medicina, Clínica Alemana-Universidad del Desarrollo, Santiago, Chile; w Department of Pediatrics, University of California San Francisco, San Francisco, CA, USA; x Department of Clinical Genetics, Churchill Hospital, Oxford, UK; y Clinical Genetics Department, Great Ormond Street Hospital for Children NHS Foundation Trust, London, UK; z Centre Référence Anomalie Développement et Syndromes Malformatifs, Centre Hospitalier Universitaire de Nice, France; aa Kariminejad-Najmabadi Pathology and Genetics Center, Tehran, Iran; ab Manchester Centre for Genomic Medicine, Institute of Human Development, Faculty of Medical and Human Sciences, University of Manchester, Manchester, UK; ac Manchester Centre for Genomic Centre for Genetic Medicine, Institute of Human Development, Faculty of Medical and Human Sciences, University of Manchester, Manchester, UK; ad St Mary's Hospital, Manchester Academic Health Science Centre, Manchester, UK; ae Department of Medical Genetics, University Medical Center Utrecht, Utrecht, Netherlands; af Institut für Humangenetik, University of Duisburg-Essen, University Hospital Essen, Essen, Germany; ag Department of Pediatrics and Translational Genetics, Academic Medical Center, University of Amsterdam, Amsterdam, Netherlands; ah Howard Hughes Medical Institutes, Houston, TX, USA; ai Epilepsy Society, Buckinghamshire, UK

## Abstract

**Background:**

Deafness, onychodystrophy, osteodystrophy, mental retardation, and seizures (DOORS) syndrome is a rare autosomal recessive disorder of unknown cause. We aimed to identify the genetic basis of this syndrome by sequencing most coding exons in affected individuals.

**Methods:**

Through a search of available case studies and communication with collaborators, we identified families that included at least one individual with at least three of the five main features of the DOORS syndrome: deafness, onychodystrophy, osteodystrophy, intellectual disability, and seizures. Participants were recruited from 26 centres in 17 countries. Families described in this study were enrolled between Dec 1, 2010, and March 1, 2013. Collaborating physicians enrolling participants obtained clinical information and DNA samples from the affected child and both parents if possible. We did whole-exome sequencing in affected individuals as they were enrolled, until we identified a candidate gene, and Sanger sequencing to confirm mutations. We did expression studies in human fibroblasts from one individual by real-time PCR and western blot analysis, and in mouse tissues by immunohistochemistry and real-time PCR.

**Findings:**

26 families were included in the study. We did exome sequencing in the first 17 enrolled families; we screened for *TBC1D24* by Sanger sequencing in subsequent families. We identified *TBC1D24* mutations in 11 individuals from nine families (by exome sequencing in seven families, and Sanger sequencing in two families). 18 families had individuals with all five main features of DOORS syndrome, and *TBC1D24* mutations were identified in half of these families. The seizure types in individuals with *TBC1D24* mutations included generalised tonic-clonic, complex partial, focal clonic, and infantile spasms. Of the 18 individuals with DOORS syndrome from 17 families without *TBC1D24* mutations, eight did not have seizures and three did not have deafness. In expression studies, some mutations abrogated *TBC1D24* mRNA stability. We also detected *Tbc1d24* expression in mouse phalangeal chondrocytes and calvaria, which suggests a role of TBC1D24 in skeletogenesis.

**Interpretation:**

Our findings suggest that mutations in *TBC1D24* seem to be an important cause of DOORS syndrome and can cause diverse phenotypes. Thus, individuals with DOORS syndrome without deafness and seizures but with the other features should still be screened for *TBC1D24* mutations. More information is needed to understand the cellular roles of TBC1D24 and identify the genes responsible for DOORS phenotypes in individuals who do not have a mutation in *TBC1D24*.

**Funding:**

US National Institutes of Health, the CIHR (Canada), the NIHR (UK), the Wellcome Trust, the Henry Smith Charity, and Action Medical Research.

## Introduction

Deafness, onychodystrophy, osteodystrophy, and mental retardation (DOOR or DOORS) syndrome (OMIM 220500) is a rare autosomal recessive disorder of unknown cause. Ronald Cantwell first described DOOR syndrome in 1975,^1^ noting that a few similarly affected individuals had been reported prior to that date. Qazi and colleagues suggested changing the name to DOORS syndrome to account for the presence of seizures in most individuals,^2^ and we use this term in this Article. The case reports of 32 affected individuals were reviewed by James and colleagues in 2007,^3^ and five others have been published since that report.^4^, ^5^, ^6^, ^7^, ^8^ Seizures, present in most patients with DOORS syndrome, usually start in the first year of life. They occasionally occur with increasing frequency or severity and are sometimes refractory to antiepileptic drugs. The seizures are often generalised tonic-clonic, but myoclonic, partial, and absence seizures also occur.^3^ Neurological involvement—apart from the epilepsy, intellectual disability, and profound sensorineural hearing loss in most affected individuals—includes occasional optic neuropathy, visual impairment, peripheral neuropathy, and abnormalities on brain MRI. The onycho-osteodystrophy affects a patient's hands and feet equally. Small or absent nails and hypoplastic terminal phalanges are seen in most individuals. A triphalangeal thumb is present in a third of affected individuals. A large base of the nose and a bulbous nose are the most common facial dysmorphisms. Cranial anomalies include microcephaly in a third of individuals and a narrow bifrontal diameter in two thirds. The rarity of DOORS syndrome, the absence of any single pathognomonic feature, the substantial clinical variability (including malformations of the brain, eyes, heart, kidneys, skeletal system, adrenal glands, and genitalia^3^), and features shared with other syndromes, make its clinical diagnosis challenging. In turn, such difficulties hinder understanding of its true prevalence and prognosis, limiting the possibility of accurate counselling. Better diagnostic measures are therefore needed.

For rare diseases in general, the identification of genetic causes has been a productive approach to improved understanding and more specific diagnosis. The genetic cause of DOORS syndrome is unknown.^9^ Strategies to identify disease-causing genes in inherited diseases have changed with the development and availability of new technologies. For example, in epilepsy, channelopathies were identified in the 1990s mostly with positional cloning and candidate gene sequencing.^10^ Next-generation sequencing, such as whole-exome sequencing, has accelerated the pace of the identification of epilepsy-associated genes. For instance, *PPRT2* mutations were identified initially in paroxysmal kinesigenic dyskinesia, and are also seen in infantile seizures and febrile seizures.^11^, ^12^ In the past year, *DEPDC5* mutations have been identified in various dominantly-inherited familial focal epilepsies.^13^, ^14^ The precise role of each protein in the CNS has not been established. In a cohort of patients with a clinical diagnosis of DOORS syndrome, we sought to identify the genetic basis by whole-exome sequencing, aiming thus to provide improved diagnostic resolution and, eventually, a better understanding of the disorder.

## Methods

### Participants

We contacted previous collaborators and physicians who had published case reports of individuals with DOORS syndrome. Patients were from 26 centres in 17 countries. Between Dec 1, 2010, and March 1, 2013, we enrolled identified individuals with DOORS syndrome who were assessed by clinical geneticists and had at least three of the five following features: deafness, abnormal nails or digits on the hands or feet, developmental delay or intellectual disability (previously known as mental retardation), and seizures.

Families were excluded if the parents could not be contacted for consent or if DNA could not be obtained from the affected individual. Further exclusion criteria were autosomal dominant inheritance and the absence of both intellectual disability and seizures, criteria that have been used elsewhere.^3^ A clinical questionnaire was completed by the collaborating physician. DNA was collected from the affected individual and both parents if possible.

A parent provided written informed consent on behalf of the affected individual. We did this study with the approval of the institutional ethics review boards of the Baylor College of Medicine (TX, USA), of University College London (London, UK), of the University of Amsterdam (Amsterdam, Netherlands), and of the University of Manchester (Manchester, UK).

### Procedures

A detailed description of the method for exome sequencing and data analysis is provided in the appendix. (p 2) Briefly, genomic DNA was fragmented, enriched for coding regions, and sequenced on an Illumina HiSeq 2000 instrument (Illumina; San Diego, CA, USA). Reads were aligned to the reference human genome, and analysed for variations from the reference. The variants were filtered to keep only rare or novel variants, and these variants were annotated for conservation data, predicted effect of the variant, variant frequency in various databases such as the Exome Variant Server, gene expression pattern, function of the gene, and phenotypes in mice and human beings. Our variant detection and filtering method was deliberately sensitive but not specific, meaning that there are multiple false-positive variants identified. False-positive variant calls can be due to artifacts introduced by the next-generation technology used,^15^ poor coverage in GC-rich regions, bases missed by next-generation sequencing because of homopolymers or dinucleotide repeats, or mapping difficulties because of gene homologues or paralogues. The most efficient method to remove these false-positive variants is to visualise alignment files (binary alignment map files), using Broad Institute's IGV viewer from affected individuals and compare them to variant files from unaffected individuals or individuals with disorders unrelated to DOORS syndrome (exomes published elsewhere^16^, ^17^, ^18^, ^19^, ^20^), which we did after restricting the candidate gene list by focusing initially on genes with an autosomal recessive inheritance pattern (one homozygous mutation or two heterozygous mutations).

We did single nucleotide polymorphism (SNP) analysis with the Illumina Infinium HD assay platform using HumanOmni1-Quad BeadChip (Illumina) according to the manufacturer's instructions, and analysed data using Illumina's GenomeStudio to look for insertions, deletions, and regions of homozygosity as well as haplotypes shared by affected siblings.

Primers used for Sanger sequencing of the genomic DNA that encodes the complete coding sequence of *TBC1D24* variant 1 (NCBI CCDS ID#55980.1) were generated with an online software ExonPrimer (see appendix p 6 for a list of primers). We generated amplicons with 10 ng of genomic DNA using TaqMan polymerase (ABI, Life Technologies, Carlsbad, CA, USA) with the manufacturer's protocol with an annealing temperature of 55°C and an amplification of 1 min. Products were sequenced by the Sanger method using the same primers, at Beckman Coulter Genomics (Danvers, MA, USA). We analysed resulting chromatograms by comparing the GenBank file for the RefSeq sequence of *TBC1D24* using Sequencher (version 4.8; Gene Codes Corporation, Ann Arbor, MI, USA).

We also did real-time PCR. We grew fibroblasts in Dulbecco's Modified Eagle's Medium with 10% fetal bovine serum, 1·5 mM glutamine, 100 IU/mL penicillin, and 50 μg/mL streptomycin (Invitrogen, Grand Island, NY, USA). Mouse tissues were from C57BL/6J wildtype mice. RNA was extracted from fibroblasts or mouse tissues using TRIzol and GlycoBlue, then cDNA was synthesised using SuperScript III First-Strand Synthesis Kit according to the manufacturer's instructions (all from Invitrogen). We did quantitative real-time PCR using the primers listed in the appendix (p 6), the FastStart DNA Master SYBR Green reagent, and a LightCycler instrument using an annealing temperature of 65°C according to the manufacturer's protocol (both from Roche NimbleGen, Madison, WI, USA).

Details of immunohistochemistry and western blotting methods used are in the appendix (pp 2–3). We generated the structural model of human *TBC1D24* with Phyre2, an online software,^21^ based on the crystal structures of the TBC domain of TBC1D1 and TBC1D4 and the TBC, LysM, Domain catalytic (TLDc) domain of the zebrafish Oxr2 protein.^22^ We made the three-dimentional figure using Pymol (version 1.6).

### Statistical analyses

We calculated differences in mRNA expression in fibroblasts using the Student's *t* test. For the expression in different mouse tissues, we did a Kruskal-Wallis one-way ANOVA on ranks, as well as a multiple comparison procedure using Dunnett's method. We used SigmaPlot (version 11.0) for all statistical analyses.

### Role of the funding source

The sponsors of the study had no role in study design, data collection, data analysis, data interpretation, or writing of the report. All authors had full access to all the data in the study and had final responsibility for the decision to submit for publication.

## Results

**Figure 1:**
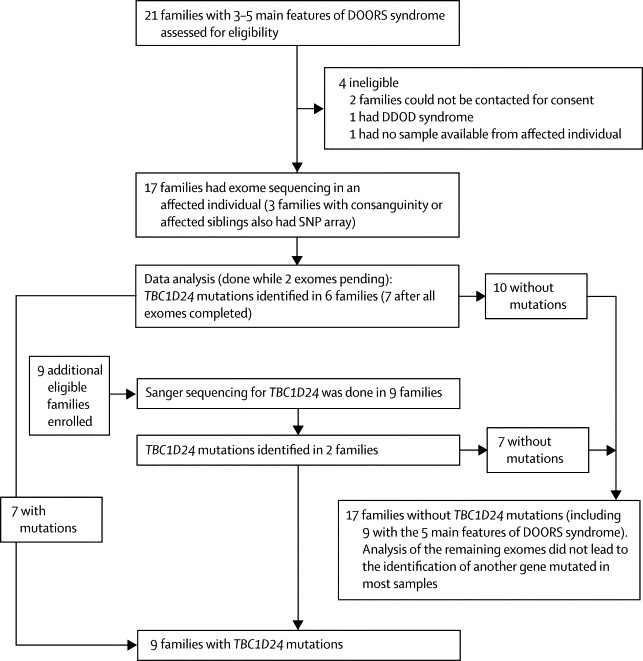
Study profile DOORS=deafness, onychodystrophy, osteodystrophy, mental retardation, and seizures. DDOD=dominant deafness-onychodystrophy.

We identified 30 families, of which four were excluded from the study: two families that could not be re-contacted for informed consent; one family in which the affected child had died and DNA was not available; and one family in which the phenotype corresponded to dominant deafness-onychodystrophy (DDOD; OMIM 124480). We did exome sequencing in the first 17 enrolled families and SNP arrays in three of these families with consanguinity or more than one affected child. Because sequencing and data preparation takes 3–5 months, we analysed exomes and SNP arrays after sequencing was completed in 15 families (figure 1). Of genes with rare or novel protein-affecting variants (compared with the frequency in the control group—ie, the 6503 exomes in the Exome Variant Server), 6645 genes had at least one rare or novel variant in at least one of fifteen samples. To restrict the list of candidate genes, we applied filtering based initially on an autosomal recessive inheritance, analysed genes mutated in several samples, and removed the false-positive variants (appendix p7). We visually compared the DOORS syndrome exome alignments to other exomes previously acquired for unrelated disorders,^16^, ^17^, ^18^, ^19^, ^20^ focusing initially on genes with a recessive inheritance pattern (one homozygous mutation or two heterozygous mutations) and variants identified in the highest number of affected individuals. Most variants in genes identified in most exomes by our annotation pipeline proved to be false-positive variants on visualisation and comparison with control exomes (appendix p 7).

We identified either homozygous or compound heterozygous mutations in *TBC1D24* (RefSeq# NM_001199107.1) in affected individuals in six of the initial 15 families in which we did exome sequencing (table 1). All mutations were novel except for the frameshift mutation, seen in the heterozygous carrier state in two of 6118 individuals sequenced for that region in the June, 2013, release of the Exome Variant Server (EVS-v.0.0.20). This server provides variant frequency data for multiple exome studies done mostly in adults with various heart and lung diseases and controls, and individual phenotypes are not available. In view of the facts that mutations in this gene have been identified as the cause of some epilepsies,^23^, ^24^, ^25^, ^26^, ^27^ that seizures are common in people with DOORS syndrome, and that all mutations we identified were novel or very rare and in some cases truncating, this gene was the most suitable candidate in our cohort. We completed exome sequencing (followed by Sanger validation) in two individuals for whom exome sequencing was already started, and did Sanger sequencing of *TBC1D24* only in the nine subsequently enrolled families (see appendix p 6 for primers). We identified three more families with *TBC1D24* mutations, one through additional exome sequencing and two by Sanger sequencing, totalling 11 affected individuals from nine families with confirmed mutations in the cohort of 26 families affected by DOORS syndrome (see table 1 for the *TBC1D24* mutations identified and the appendix p 4 for analyses done on each family). Annotations for all *TBC1D24* variants (from DOORS syndrome, the other associated epileptic disorders, and the Exome Variant Server), including population frequency, conservation scores and PolyPhen2 scores are in the appendix (p 8). Collectively, other coding variants in *TBC1D24* were seen in the heterozygous state in 343 (5%) of 6503 of the Exome Variant Server cohort.

**Table 1:** Mutations identified in *TBC1D24*

	**Origin**	**Maternal allele (DNA)**	**Maternal allele (protein)**	**Paternal allele (DNA)**	**Paternal allele (protein)**
Individual 1	Japan	724C→T	Arg242Cys	118C→T	Arg40Cys
Individual 2a	USA	724C→T	Arg242Cys	724C→T	Arg242Cys
Individual 2b	USA	724C→T	Arg242Cys	724C→T	Arg242Cys
Individual 3	Germany	1008delT	His336GlnfsTer12*	1206+5G→A	Splicing
Individual 4	India	724C→T	Arg242Cys	724C→T	Arg242Cys
Individual 5a	Chile	58C→G	Gln20Glu	724C→T	Arg242Cys
Individual 5b	Chile	58C→G	Gln20Glu	724C→T	Arg242Cys
Individual 6	France	1008delT	His336GlnfsTer12*	Not identified	Not identified
Individual 7	Brazil	724C→T	Arg242Cys	724C→T	Arg242Cys
Individual 8	Turkey	119G→T	Arg40Leu	119G→T	Arg40Leu
Individual 9	UK	328G→A	Gly110Ser	999G→T	Leu333Phe

Individuals with letters a and b after the number are siblings·

* The histidine (His) at position 336 is replaced by a glutamine (Gln), which is followed by a frameshifted protein sequence (fs) ending with a termination codon (Ter) after 12 aminoacids.

**Figure 2:**
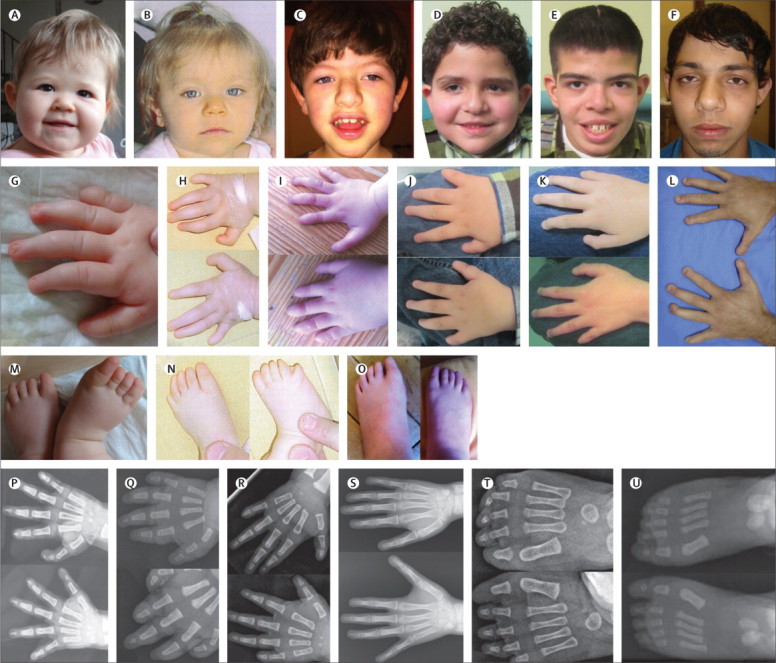
Physical features of participants with *TBC1D24* mutations (A–F) Some individuals have a wide base of the nose and bulbous end of the nose (individual numbers from tables corresponding to each panel: A=3, B=6, C=5a, D=2b, E=2a, F=7). (G–L) Hands in individuals with *TBC1D24* mutations have triphalangeal thumbs, brachydactyly, short terminal phalanges, and hypoplasia or aplasia of the nails (G=3, H=6, I=5a, J=2b, K=2a, I=7). (M–O) Feet with short terminal phalanges and hypoplasia or aplasia of the nails (M=3, N=6, O=5a). (P–S) Radiographs of the hands in individuals with *TBC1D24* mutations. Note the triphalangeal thumbs and the short terminal phalanges (P=4, Q=5a, R=6, S=1). (T,U) Radiographs of the feet in individuals with *TBC1D24* mutations, showing short terminal phalanges (T=6, U=5a).

**Figure 3:**
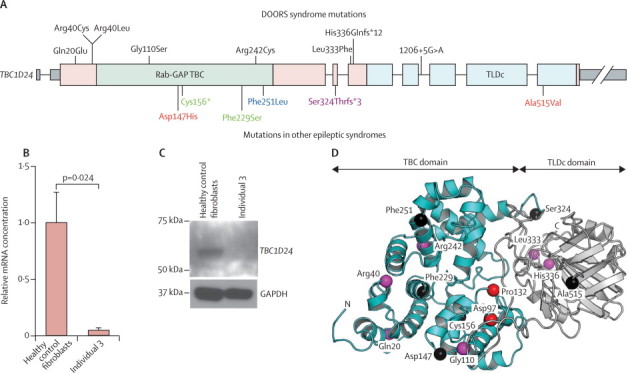
Mutations in *TBC1D24* (A) Location of the mutations identified in DOORS syndrome and in other epilepsy syndromes. Phe251Leu (blue; homozygous mutation, affecting four siblings) causes focal epilepsy, dysarthria, intellectual disability, cortical thickening, cerebellar atrophy.^23^, ^24^ Asp147His and Ala515Val (red; compound heterozygous mutation, affecting seven individuals in one family) causes familial infantile myoclonic epilepsy.^25^ Ser324Thrfs*3 (purple; homozygous mutation, affecting five individuals in one family) causes myoclonic epilepsy, dystonia, hemiparesis, autonomic signs, lethargy, progressive diffuse cerebral atrophy.^27^ Phe229Ser and Cys156* (green; compound heterozygous mutation affecting two siblings)—causes familial malignant migrating partial seizures of infancy, progressive diffuse cerebral atrophy.^26^ The diagram also shows the exonic structure of *TBC1D24,* with the introns not drawn to scale. (B) Real-time PCR of *TBC1D24* in fibroblasts from the individual with a frameshift deletion and a splicing mutation, showing substantial reduction of *TBC1D24* mRNA in affected fibroblasts. (C) Western blot analysis of the cells used in panel B, showing that TBC1D24 protein is undetectable by this method in affected fibroblasts. (D) Structural model of TBC1D24 with the TBC domain coloured in blue and the TBC, LysM, Domain catalytic (TLDc) domain coloured in grey. The N terminal and C termini are labelled, and the red spheres show the alpha carbon atoms of the residues aligning with the arginine and glutamine fingers interacting with the GTP of Rab proteins in other TBC proteins,^40^ based on the structure of Gyp1p in complex with Rab33.^37^ The carbon atoms of residues substituted in DOORS syndrome are shown with purple spheres and those substituted in other epilepsy syndromes are shown with black spheres.

We saw a wide variety of seizure types in our cohort of individuals with *TBC1D24* mutations, including generalised tonic-clonic, complex partial, focal clonic seizures, and infantile spasms. Table 2, Table 3 list the clinical features of individuals with and without *TBC1D24* mutations. Figure 2 shows photographs of the face, hands, feet, and radiographs. The mutations are listed in table 1 and shown in figure 3. The conservation of the affected residues and nucleotides is given in the appendix (p 17). In families with *TBC1D24* mutations and consanguinity, the mutations were homozygous and *TBC1D24* was in a region of homozygosity as identified by SNP array or homozygosity mapping from exome data. The regions of homozygosity are given in the appendix (p 11).

**Table 2:** Clinical features of patients with DOORS with the *TBC1D24* mutations

	**Origin**	**Age in March, 2013**	**Sex**	**Consanguinity**	**Abnormal finger nails**	**Abnormal fingers**	**Triphalangeal thumb**	**Abnormal toenails**	**Abnormal toes**	**Developmental delay or intellectual disability**	**Feeding difficulties**	**Deafness**	**Urine 2-oxoglutaric acid**	**Seizures**	**Age of seizure onset and pharmacoresponsiveness**	**Brain imaging**	**Cranial shape anomalies**	**Growth parameters (percentiles)**	**Other findings and reference if published**
1	Japan	21 years	M	N	Y	Y	Y	N	N	Y	N	Y	Normal	Absence, GTCS	2 months, moderate control with valproic acid, zonisamide, clonazepam, carbamazepine	Thin cerebellar cortex, hyperintense on T2-imaging, myelination delay	N	At 11 years: height 135 cm (10th), weight 31 kg (20th), head 52·5 cm (25th)	Autism spectrum disorder^7^
2a	USA	15 years	M	N	Y	Y	N	Y	Y	Y	Y	Y	Increased	Complex partial	6 months, good control with topiramate, lamotrigine, lacosamide	Normal MRI	Sagittal craniosynostosis	At 14 years: height 154 cm (10th), weight 51 kg (50th), head 54·5 cm (40th)	Large central incisors, widely spaced teeth, delayed eruption of permanent teeth, calcaneal deformity, myopia
2b	USA	8 years	M	N	Y	Y	N	Y	Y	Y	Y	Y	Increased	Complex partial	4 months, good control with topiramate, clorazepate, lacosamide	Punctate foci of increased T2 signal in right frontal region Increased FLAIR signal around occipital horn	N	At 7 years: height 111 cm (2nd), weight 35 kg (98th), head 52 cm (25th)	Double outlet right ventricle, myopia
3	Germany	2·5 years	F	N	Y	Y	N	Y	Y	Y	N	Y	Increased	Focal, secondarily GTCS	6 weeks, poor control, at least 12 AED tried*	Delayed myelination	N	At 13 months: length 82 cm (97th), weight 12 kg (80th), head 42·5 cm (<3rd)	Microcephaly, nephrocalcinosis, myopia
4	India	3·5 years	M	Y	Y	Y	Y	Y	Y	Y	N	Y	Increased	Focal clonic	5 months, good control with valproate and topiramate	Normal MRI	N	At 14 months: height 65 cm (<3rd), weight 6 kg (<3rd), head 43·5 cm (<3rd)	Symmetrical growth retardation^4^
5a	Chile	9 years	M	N	Y	Y	N	Y	Y	Y	Y	Y	Normal	Complex partial	3 months, good control with phenobarbital and clobazam	Normal MRI	Brachycephaly	At 6 years 8 months: height 120 cm (50th), weight 25 kg (75th), head 53 cm (75th)	Widely spaced teeth
5b	Chile	1 years	M	N	Y	Y	N	Y	Y	Y	N	Y	Normal	Complex partial	N/A	Normal MRI	N	At birth: length 49 cm (35th), weight 3 kg (20th), head 33·5 cm (15th)	None
6	France	1 years	F	N	Y	Y	N	Y	Y	Y	N	Y	Increased	GTCS, focal clonic	3 months, moderate control with clonazepam, valproic acid, topiramate	Normal MRI	N	At 2 years 2 months: length 85 cm (25th), weight 10·7 kg (10th), head 44 cm (<3rd)	Microcephaly. Mother had absence seizures as a child
7	Brazil	22 years	M	N	Y	Y	N	Y	Y	Y	N	Y	Normal	Infantile spasms, absence, GTCS	7 months, good control with carbamazepine and clobazam	Hyperintense T2 signals in the cerebellar hemispheres, especially on the left	N	At 22 years: height 170 cm (25th), weight 64 kg (25th), head 54 cm (5th)	Hypothyroidism
8a	Turkey	Died at 6 months	F	Y	Y	Y	Y	Y	Y	Y	N	Y	N/A	Myoclonic, complex partial	2 months, moderate control with phenobarbital	Normal cranial ultrasound after birth	Prominent occiput, frontal bossing, bitemporal narrowing	At 3·5 months: height 59 cm (25th), weight 5·2 kg (20th), head 40·7 cm (50th)	Capillary haemangioma, broad tip of the nose Narrow palate, broad alveolar ridge, short frenulum Died after an epileptic attack
8b	Turkey	Died at 9 months	M	Y	Y	Y	N	Y	Y	Y	N	Y	N/A	Myoclonic	2 months, moderate control with clonazepam, phenytoin, valproic acid, phenobarbital, diazepam	Initial MRI normal, subdural effusion and cortical atrophy at 4 months	N	At 4·5 months: height 63 cm (30th), weight 7 kg (50th), head 42 cm (30th)	Broad tip of the nose, high palate, broad alveolar ridge No response to light Died after an epileptic attack
9	UK	2·5 years	M	N	Y	Y	N	Y	Y	Y	N	Y	Increased	GTCS, multifocal myoclonic jerks	9 weeks, moderate control with valproic acid and levetiracetam	Normal MRI	Asymmetric brachycephaly	At 7 months: height N/A, weight 7·6 kg (15th), head 43·2 cm (15th)	Left kidney hydronephrosis, high arched palate

Patients listed by each individual's unqiue identifier. N/A=not available. Y=yes. N=no. M=male. F=female. ASD=atrial septal defect. GTCS=generalised tonic-clonic seizures. Head=head circumference. Individuals with letters a and b after the number are siblings. Recent cognitive or developmental assessments were not available for most individuals, but the global development was estimated for individual 8a as that of a 6-week-old baby when she was 12 weeks old, for individual 5a as that of a 1-year-old boy when he was 3 years old, and individual 1 had a developmental quotient of 55 with autism spectrum disorder when he was 4 years old. Hearing loss was qualified as profound sensorineural hearing loss in individuals 1, 2b, 3, 4, and 6. Cochlear implants have been beneficial in individual 3.

* Antiepileptic drugs used or tried with limited success in individual 3 include oxcarbazepine, clobazam, levetiracetam, valproic acid, lamotrigine, sulthiame, diazepam, clonazepam, midazolam, zonisamide, and cortisone pulses. In family 8, DNA was not available from individual 8b, only for 8a and parents. Sensorineural hearing loss was unilateral in individual 12a; in individual 15, it was severe on the right and moderate on the left.

**Table 3:** Clinical features of patients with DOORS without the *TBC1D24* mutations

	**Origin**	**Age in March, 2013**	**Sex**	**Consanguinity**	**Abnormal finger nails**	**Abnormal fingers**	**Triphalangeal thumb**	**Abnormal toe nails**	**Abnormal toes**	**Developmental delay or intellectual disability**	**Feeding difficulties**	**Deafness**	**Urine 2-oxoglutaric acid**	**Seizures**	**Age of seizure onset and pharmacoresponsiveness**	**Brain imaging**	**Cranial shape anomalies**	**Growth parameters (percentiles)**	**Other findings and reference if published**
10	USA	4years	F	N	Y	Y	N	Y	Y	Y	Y	Y	Increased	GTCS, complex partial	8 months, good control with leviteracetam	Delayed myelination in newborn period, normal at 3 years	N	At 4 years: height 84 cm (<3rd), weight 10·9 kg (<3rd)	Coloboma left retina, coarctation of the aorta, hip dysplasia, tethered cord, sacral dimple, small and low-set ears, wide nasal bridge, slow hair growth
11	Brazil	12years	M	N	Y	Y	N	Y	Y	Y	N	Y	Increased	No seizures	N/A	Dolichocephaly	N	At 12 years: height 145 cm (25th), weight 44·8 kg (65th), head 54·5 cm (60th)	Micrognathia, bilateral epicanthal folds, anteverted nares, high-arched palate
12a	Belgium	Died at 3 years	F	N	Y	Y	Y	Y	Y	Y	Y	Y	Increased	GTCS	3 months, resistant to treatment	Hyperintensities in the pons	Metopic ridge, biparietal narrowing	At 3 months: height 3rd, weight 3rd, head 10–25th	Metopic ridge, biparietal narrowing, unilateral hearing loss Short fixation, abnormal eye movements, and intermittent strabismus, aspiration pneumonias, dysplastic ears^9^
12b	Belgium	5years	M	N	Y	Y	Y	Y	Y	Y	Y	Y	Increased	Myoclonic	3 weeks, poor control	Thin corpus callosum	N	At birth, normal growth parameters	At 2 months, VEP showed a weak response, and ERG was normal. Patent ductus arteriosus, dysplastic ears^9^
13	India	Died at 3months	F	Y	Y	Y	Y	Y	Y	Y	N	N	N/A	No seizures	N/A	N/A	Broad forehead	At birth: 2·2 kg (<3rd). At 2·5 months: head 33 cm (<3rd)	Microcephaly, progeroid appearance, bilateral low-set ears, dysplastic pinna, high arched palate, macrostomia, submucous clefting of palate, protruding tongue, anteverted nares, long smooth philtrum, hypertrichosis, broad forehead
14	Netherlands	2·5years	F	N	Y	Y	N	N	N	Y	Y	Y	Normal	No seizures	N/A	N/A	N	At 10 months: height 69 cm (15th), weight 6·6 kg (<3rd), head 42·5 cm (5th)	VSD, long eyelashes, cleft palate, cup-shaped ears
15	India	8years	F	N	Y	Y	N	Y	Y	Y	N	Y	N/A	Focal motor	4 years, good control	Normal MRI	High forehead	At 4·5 years: height <3rd, weight <3rd, head <3rd	Microcephaly, horizontal nystagmus
16	UK of African origin	6years	M	N	Y	Y	N	Y	Y	Y	Y	Y	N/A	GTCS	2 years 9 months; good control with valproic acid	Partial corpus callosum agenesis Small lesion in right putamen suggestive of a developmental venous anomaly	N	At 3 years 3 months: weight 15·6 kg (65th), height 93·1 cm (15th), head 48·1 cm (5th)	Severe gastro-oesophageal reflux, ASD requiring surgical repair, coarse facial features, low frontal hairline, webbed neck, micrognathia, midline groove of lower lip, thick lips, thickened gingiva, long tongue, anteverted abnormally formed ears Severe kyphoscoliosis (congenital) and calcaneovalgus Tracheomalacia, tracheostomy, multiple keloid scar formation
17	Lebanon	1year	F	N	Y	Y	N	Y	Y	Y	Y	Y	N/A	No seizures	N/A	Aplasia of falx cerebri	N	At 11 weeks: length 57 cm (8th), weight 4·5 kg (3rd), head 36 cm (<3rd)	High frontal and temporal hairline, hypertelorism, upslanting palpebral fissures, broad nose, low-set and posteriorly rotated ears Hypoplastic patellae Uterus bicornis, hypercalcaemia
18	Iran	14years	M	Y	Y	Y	N	N	N	Y	N	Y	N/A	GTCS	1 year, good control with phenobarbital	N/A	Brachycephaly	At 14 years: head 50·5 cm (<3rd)	Multicystic left kidney, hypertrophic right kidney, cleft palate, bilateral inguinal hernia, short PF, blepharophimosis, microcornea, telecanthus, prominent nose, bilateral narrowing of ear canals, small mouth, malocclusion of teeth, hallux valgus Affected sibling died at 1·5 years
19	USA	Died at 3weeks	M	N	Y	Y	N	Y	Y	Y	N	Y	Normal	No seizures	N/A	Dandy-Walker malformation, agenesis of the corpus callosum	N	At birth: length 44 cm (<3rd), weight 2·26 kg (<3rd), head 33 cm (17th)	Blindness, widely spaced nipples, low-set ears
20	Canada	2years	F	N	Y	Y	N	Y	Y	Y	Y	Y	Normal	No seizures	N/A	Dandy-Walker malformation, small focal area of restricted diffusion in the right posterior basal ganglia area	Large anterior fontanelle, microcephaly, narrow bifrontal diameter	At 1 year: height <3rd, weight <3rd, head <3rd	Sparse fine hair, right optic nerve morning glory anomaly, persistent left superior vena cava draining into the coronary sinus, atrial septal defect, mild tricuspid regurgitation, laryngomalacia, short sternum, right thoracic scoliosis, low conus medullaris, rocker bottom feet, widely spaced nipples, high arched palate, short neck
21	Ireland	Died at 10years	M	N	Y	N	Y	Y	N	Y	Y	Y	Normal	GTCS, absence, myoclonic	6 months	Normal MRI	Dolichocephaly	At 3 years 6 months: height 110 cm (>97th), weight 19 kg (95th), head 58 cm (>97th)	Double outlet right ventricle, recurrent chest infections, supernumerary nipple, broad alveolar ridge, blindness, hydronephrosis
22	UK of Indian origin	29years	F	N	Y	N	N	Y	N	Y	Y	N	Normal	GTCS, Complex partial, episodes of status epilepticus	3 months, moderate control with valproate, levetiracetam, pregabalin, phenytoin, topiramate	Diffuse atrophy cerebrum and cerebellum, asymmetric lateral ventricles	N	At 29 years: weight 50 kg (15th)	Long and thin face, high arched palate
23	India	7years	M	Y	Y	Y	Y	Y	N	Y	N	N	Normal	GTCS	11 months, good control with phenytoin and phenobarbital	Normal MRI	N	At 6 years: height 110 cm (15th), weight 18 kg (15th), head 46·5 cm (<3rd)	Delayed permanent dentition, small teeth, cavities, anteverted nares, low-set pinna, open mouth, ASD, sibling with similar phenotype died at 1 year 9 months
24	Poland	22years	M	Y	Y	Y	Y	Y	Y	Y	N	Y	Normal	No seizures	N/A	N/A	N	At birth: length 57 cm (>97th), height 3·9 kg (75th), head 32 cm (4th)	Delayed teething, haemangioma, epicanthus of the left eye, ptosis, strabismus, asymmetric face, high-arched palate^8^
25	New Zealand	12years	M	N	Y	Y	Y	Y	Y	Y	Y	Y	Normal	Infantile spasms	1 year, good control with vigabatrin	N/A	N	At 7 years: height 188 cm (>97th), weight 24 kg (60th)	Broad nose, dental misalignment and irregularities, ASD
26	UK	15years	M	N	Y	Y	Y	Y	Y	Y	N	Y	Normal	No seizures	N/A	N/A	N	At 5 months: length 64·5 cm (25th), weight 6·3 kg (5th), head 44·5 cm (75th)	Premature birth

Patients listed by each individual's unqiue identifier. N/A=not available. Y=yes. N=No. M=male. F=female. ASD=atrial septal defect. GTCS=generalised tonic-clonic seizures. Head=head circumference. VEP=Visual evoked potential. VSD=ventricular septal defects. ERG=electroretinogram. PF=palpebral fissures. Individuals with letters a and b after the number are siblings. Recent cognitive or developmental assessments were not available for most individuals. Intellectual disability or developmental delay was categorised as mild for individual 24, moderate for individual 14, and severe for individuals 10, 11, 12a, 12b, 16, 21, 22, 23, and 25. Formal audiometric test results were not available for most individuals. Sensorineural hearing loss was unilateral in individual 12a; in individual 15, it was severe on the right and moderate on the left.

Because of the recessive nature of the disease and the presence of mutations predicted to lead to premature protein termination, we proposed that the mutations cause a loss of TBC1D24 function. We assessed this hypothesis in fibroblasts from a biopsy previously done for clinical reasons in individual 3, who was compound heterozygote for a splice site mutation and a frameshift mutation in *trans*. This frameshift mutation, because it is not in the last exon, is predicted to lead to nonsense-mediated decay (NMD) of the mRNA.^28^ The splice donor mutation after exon five, because it is located five nucleotides away from a splice site (1206+5G→A), might or might not affect splicing efficiency. If it affects splicing efficiency, the mutation would lead to an aberrant protein-encoding mRNA, thus engaging NMD. To test for NMD, we did real-time PCR (figure 3). *TBC1D24* mRNA concentrations in the fibroblasts from individual 3 were 5% (SE 1%) of the concentrations from three unaffected controls (Student's *t* test p=0·024), confirming that the mRNA from both alleles undergoes NMD and thus showing a loss of *TBC1D24* function. TBC1D24 protein from these fibroblasts could not be detected by western blot analysis (figure 3).

We next assessed *Tbc1d24* tissue expression. *Tbc1d24* is known to be widely expressed, most highly in the brain (especially in pyramidal neurons), kidneys, and salivary and lacrimal glands.^29^, ^30^ In the brain, the regions with the highest expression levels are the hippocampus and the somatomotor areas of the isocortex (appendix p 13). Using an antibody against TBC1D24, we studied expression in digital chondrocytes, in view of the distal phalangeal hypoplasia in DOORS syndrome and the high expression seen in chondrocytes in the Human Protein Atlas. Expression was high in the chondrocytes of the distal phalanges of the forelimbs (appendix p 19). Moreover, we assessed expression by real-time PCR in various newborn mouse tissues and noticed high expression in the calvarium (appendix p 19), which correlates well with the cranial shape phenotype and occasional cranial synostosis seen in affected individuals (table 2).

TBC1D24 contains a TLDc domain and regulates Rab proteins. In our cohort, we identified no mutations in the other TLDc domain-containing genes by exome sequencing (appendix p 14). By homology modelling, the substitutions we identified did not seem to affect portions of the protein predicted to interact with the TLDc domain, nor were they in the region that typically interacts with GTP in other TBC proteins (figure 3). Because TBC1D24 regulates Rab proteins and because of clinical overlap between DOORS syndrome and Martsolf syndrome, we also assessed *RAB3GAP2*. No *RAB3GAP2* mutations were identified in families without *TBC1D24* mutations (appendix p 14). Moreover, in genetic locus mapping by SNP arrays, or homozygosity mapping from exome data in some families without *TBC1D24* mutations, we did not identify a mutated gene in common in these families (details on the regions identified are given in the appendix p 11). Analyses are ongoing to identify candidate genes with mutations in families following either a de novo dominant or a recessive inheritance pattern. Details on analysis in the ten exomes without *TBC1D24* mutations are given in the appendix (pp 15–16).

Some individuals without *TBC1D24* mutations had typical features of DOORS syndrome, including the more specific signs of triphalangeal thumbs and 2-oxoglutaric aciduria, and were clinically indistinguishable from those with *TBC1D24* mutations. This finding suggests genetic heterogeneity in DOORS syndrome. In the individuals without *TBC1D24* mutations, eight did not have seizures and three did not have deafness (including one without seizures or deafness) and some individuals had additional malformations (eg, Arnold-Chiari malformation), which suggests that our cohort might include some individuals with disorders that overlap with but are different from DOORS syndrome (table 2). All individuals with *TBC1D24* mutations had all five features making the DOORS acronym. If we stratify based on this strict definition of DOORS syndrome, *TBC1D24* mutations were seen in nine of 18 families.

## Discussion

We have identified mutations in *TBC1D24* as a probable cause of DOORS syndrome. Previously reported, but different, mutations in *TBC1D24* cause various epileptic syndromes. A homozygous Phe251Leu substitution in four siblings of an Arab-Israeli family caused focal epilepsy, dysarthria, mild to moderate intellectual disability, and cortical thickening with cerebellar atrophy and a strong signal in the ansiform lobule of the cerebellum on T2-weighted and fluid-attenuated inversion recovery MRI.^24^ In an Italian family, compound heterozygous substitutions (Asp147His and Ala515Val) cause familial infantile myoclonic epilepsy (FIME [OMIM 605021]) in seven individuals with normal intellect and normal MRI, except for periventricular nodular heterotopia in one individual.^25^ A homozygous truncating mutation (969_970delGT, Ser324Thrfs*3) in exon 3 was identified in five Turkish individuals with a recessive form of myoclonic epilepsy with episodic dystonia, hemiparesis, autonomic signs, and lethargy.^27^ The five Turkish individuals developed the following: chronic dystonia, progressive diffuse cerebral atrophy, and early death. Exon 3 is spliced out in isoform 2, which is expressed predominantly in non-neural tissues. Finally, two compound heterozygous substitutions (Phe229Ser and Cys156*) were identified in two siblings with familial malignant migrating partial seizures of infancy, with progressive diffuse atrophy of the grey matter sparing the posterior fossa and early death.^26^ The diversity of seizure types seen in *TBC1D24*-associated epileptic syndromes and in DOORS syndrome is striking (table 2), and might point to a general epileptogenic mechanism. None of the patients in previous reports of *TBC1D24* mutations had digital anomalies or deafness, and none of the above mutations was identified in our DOORS syndrome cohort, showing a clear genotype-to-phenotype correlation. The reason why some mutations cause DOORS syndrome and others cause only epilepsy might lie partly in the way the mutations affect interaction patterns with protein partners. This idea will need to be investigated. Some genes implicated in epilepsy seem capable of causing a wide variety of types of epilepsy, with greater or fewer additional neuropsychiatric features, and others have been associated with brain malformations or complex dysmorphic syndromes.^31^ However, phenotypic pleiotropy has rarely been reported to span the spectrum from seizures alone (eg, previous reports on *TBC1D24* mutations) to multi-systemic syndromic disorders such as DOORS syndrome. These emerging findings of both genotype-phenotype complexity and genetic pleiotropy lend support to the view that, with accumulating mutational data, the fuller coverage afforded by exome (or genome) sequencing might be more useful than targeted gene panels in clinical practice.

Five of the families with DOORS syndrome in our study have substitutions affecting the arginine at position 242, and two have substitutions affecting the arginine at position 40, suggesting that these residues are crucial in TBC1D24 function. Both are in a CpG island and affect CG dinucleotides. CpG nucleotides are more mutation-prone, which might explain mutation recurrence in individuals of different ethnic origins. Two patients also shared another recurrent mutation unique to DOORS (His336Glnfs*12).

TBC1D24 is a member of the Tre2–Bub2–Cdc16 (TBC) domain-containing RAB-specific GTPase-activating proteins, which coordinate Rab proteins and other GTPases for the proper transport of intracellular vesicles. TBC1D24 is the only TBC/RabGAP with a TLDc domain, which is thought to be involved in oxidative stress resistance and to have catalytic activity for unknown substrates.^32^, ^33^ The TLDc domain is also seen in the proteins encoded by the human genes *OXR1*, *NCOA7*, *KIAA1609*, and *C20orf118*, which do not share known functions. Mice without *Oxr1* have oxidative stress-induced neurodegeneration,^34^ which suggests a possible link with the neurodegeneration seen in some individuals with *TBC1D24* mutations.^27^ We saw no mutations in other TLDc domain encoding genes in this study. Several diseases have been associated with other aberrant Rab proteins and Rab-associated proteins. Some clinical overlap exists between DOORS syndrome and Martsolf syndrome (caused by mutations in *RAB3GAP2* gene [OMIM 212720]; shared aspects include seizures, intellectual disability, abnormal toenails, and short phalanges), and other syndromes. We identified no mutations in *RAB3GAP2*.

Further research is needed to establish the precise role of TBC1D24 not only in the nervous system, but also in the skeletal system and other systems affected in DOORS syndrome. In *Caenorhabditis elegans*, C31H2.1 (a *TBC1D24* orthologue) was implicated in synaptic function by an RNAi screen.^35^ In Drosophila, the orthologue Skywalker (Sky) enables endosomal trafficking in synaptic vesicles by facilitating GTP hydrolysis by Rab35, thus controlling synaptic vesicle rejuvenation and neurotransmitter release.^36^ Human TBC1D24 has low similarity to the Drosophila protein^36^ and neither possesses the arginine and glutamine residues (the so-called RQ fingers) that are crucial to catalyse GTP hydrolysis by Rab proteins.^37^ Whether human TBC1D24 is able to also facilitate Rab protein-mediated GTP hydrolysis is not known.

Our findings implicate defective vesicular trafficking as the possible basis of the complex phenotype in individuals with DOORS syndrome in view of findings from previous studies in other TBC proteins (panel). The seizure phenotype present in all individuals with *TBC1D24* mutations, and the studies in Drosophila, suggest a potential role for aberrant neurotransmitter release in the neurological manifestations of the disease. However, further studies in model organisms will be needed to study this in detail. At present, we are generating transgenic mice that will help us to assess these possibilities.

Panel **Research in context**

**Systematic review**

We searched PubMed and the references of included papers for articles published from Jan 1, 1970, to March 1, 2013. We used the following search terms: “DOOR syndrome”, “DOORS syndrome”, “deafness and onychodystrophy”, “*TBC1D24*”, “2-oxoglutaric aciduria”, “2-oxoglutarate”, “epilepsy”, and “exome sequencing”. We retrieved all previous reports of individuals with DOORS syndrome and assessed the key clinical features, which present in most patients as sensorineural deafness, nail hypoplasia, terminal phalangeal hypoplasia, triphalangeal thumbs, developmental delay, intellectual disability, seizures, craniofacial anomalies, 2-oxoglutaric aciduria, and MRI anomalies. 25% or more of patients have consanguinity or affected siblings, and optic atrophy. Fewer than 25% of patients have congenital heart defects, urinary tract anomalies, and peripheral neuropathy. Genetic testing in the one previous study excluded the candidate genes *BMP4* and *OGDH.*

**Interpretation**

Our findings suggest that mutations in *TBC1D24* are a key genetic cause in some individuals with DOORS syndrome, and imply that testing for *TBC1D24* mutations should be considered by clinicians if they suspect a diagnosis of DOORS syndrome. Our findings also substantiate the role of this gene in various epilepsy syndromes, as is seen also with other epilepsy-related genes such as *SCN1A*, *KCNQ2*, and *PRRT2*. Moreover, our findings lend support to the idea of pleiotropy in epilepsy genetics because, although some mutations in *TBC1D24* can cause mild epilepsy without other substantial associated features, other mutations in *TCB1C24* cause epilepsy as part of a syndrome with features beyond the nervous system. Our findings lend support to the idea that the genomic coverage provided by whole-exome sequencing is likely to prove useful in the diagnosis of rare neurological diseases that have clinical and genetic heterogeneity.

Exome sequencing, although a powerful method to identify new genes linked to mendelian disorders, does have limitations. In a medical genetics clinical setting, exome sequencing has a diagnostic yield of 20–35%.^38^, ^39^ Some limitations are linked to the strategy of sequencing only exons: promoter mutations or deep intronic mutations affecting splicing will be missed. Other limitation are inherent to our study design; we did not analyse oligogenic inheritance (combination of one mutated allele in each of two or more genes) or synonymous exonic variants, which could be potentially deleterious. Finally, some limitations are technical—eg, GC-rich regions are difficult to sequence and map, coverage can vary from sample to sample, and reads in genes that have paralogues or homologues can also be difficult to map to the reference genome. Future strategies to identify the causative genes in individuals with DOORS syndrome but without *TBC1D24* mutations could include consideration of oligogenic inheritance models, analysis of output for multiple different genes in the same pathway, an increase in enrolment and number of exomes or depth of exome sequencing, whole-genome sequencing, and analysis of exome data for copy-number variants.

The phenotypic similarity between patients with and without *TBC1D24* mutations is highly suggestive of genetic heterogeneity in DOORS syndrome. In this study, individuals with *TBC1D24* mutations all had the five features of the DOORS acronym. However, other features were greatly variable, in terms of seizure types, pharmacoresponsiveness, brain-imaging abnormalities, cranial shape, 2-oxoglutaric aciduria, a triphalangeal thumb, and growth parameters. We saw a similar pattern in individuals without *TBC1D24* mutations: nine had the five features making the DOORS acronym; several had 2-oxoglutaric aciduria or a triphalangeal thumb. Further discussion on 2-oxoglutaric aciduria is provided in the appendix (p 20).

Through a combination of careful clinical phenotyping and exome sequencing, we have identified the molecular basis of DOORS syndrome in about a third of individuals included in our cohort, or in half of the 18 families in which affected individuals had the five features making the DOORS acronym. We suggest that individuals without deafness and seizures but with the other features should still be screened for *TBC1D24* mutations because we are only beginning to understand the genetic causation of DOORS syndrome: the discovery of *TBC1D24* mutations in a patient with an appropriate phenotype confirms the diagnosis of DOORS syndrome, but more cases need to be studied to establish the full clinical use of *TBC1D24* mutation testing. As has occurred with other rare diseases for which genetic analysis has elucidated the cause, we hope that this gene discovery can help galvanise clinical and scientific progress in understanding and treating DOORS syndrome.
