# Supplementary material for: The genetic basis of DOORS syndrome: an exome-sequencing study
Source: Lancet Neurol. 2014 Jan;13(1):44–58. doi: 10.1016/S1474-4422(13)70265-5 (PMC3895324; doi:10.1016/S1474-4422(13)70265-5)
Supplement: Supplementary appendix [file mmc1.pdf]

THE LANCET Neurology

Supplementary webappendix

This webappendix formed part of the original submission and has been peer reviewed. We post it as supplied by the authors.

Supplement to: Campeau PM, Kasperaviciute D, Lu JT, et al. The genetic basis of DOORS syndrome: an exome-sequencing study. *Lancet Neurol* 2013; published online Nov 29. http://dx.doi.org/10.1016/S1474-4422(13)70265-5.

Supplementary Data for:

The genetic basis of DOORS syndrome: an exome sequencing study.

Philippe M Campeau, Dalia Kasperaviciute, James T Lu, Lindsay C Burrage, Choel Kim, Mutsuki Hori, Berkley R Powell, Fiona Stewart, Têmis Maria Félix, Jenneke van den Ende, Marzena Wisniewska, Hülya Kayserili, Patrick Rump, Sheela Nampoothiri, Salim Aftimos, Antje Mey, Lal D.V. Nair, Michael L Begleiter, Isabelle De Bie, Girish Meenakshi, Mitzi L. Murray, Gabriela M Repetto, Mahin Golabi, Edward Blair, Alison Male, Fabienne Giuliano, Ariana Kariminejad, William G Newman, Sanjeev S Bhaskar, Jonathan E Dickerson, Bronwyn Kerr, Siddharth Banka, Jacques C Giltay, Dagmar Wieczorek, Anna Tostevin, Joanna Wiszniewska, Sau Wai Cheung, Raoul C. Hennekam, Richard A Gibbs, Brendan H Lee, Sanjay M Sisodiya.

Contents

Supplementary Methods. Exome sequencing and analysis, Immunohistochemistry and Western blotting. 2
Supplementary Table 1. Genetic analyses performed for each affected individual. 4
Supplementary Table 2. Primers used for Sanger sequencing and real-time PCR. 6
Supplementary Table 3. Table illustrating the process by which we identified recurrent mutations in TBC1D24 in the cohort of individuals with DOOR/S syndrome. 7
Supplementary Table 4. Annotations for all known variants in *TBC1D24*. 8
Supplementary Table 5. Candidate regions from SNP array or homozygosity mapping of exome data. 11
Supplementary Table 6. Review of the expression data on *TBC1D24* in human and mouse tissues, detected at the level of mRNA or protein, from publications or public databases. 13
Supplementary Table 7. Coverage data for candidate genes. 14
Supplementary Table 8. Candidate gene analysis from exome data in 10 individuals without *TBC1D24* mutations. 15
Supplementary Table 9. Gene names for recessive model from exome data in 10 individuals without *TBC1D24* mutations. 16
Supplementary Figure 1. Conservation across species of the residues affected by missense substitutions. 17
Supplementary Figure 2. Additional *Tbc1d24* expression data from public databases. 18
Supplementary Figure 3. Tbc1d24 expression profiling. 19
Supplementary Discussion. 2-Oxoglutaric aciduria and DOORS syndrome types. 20
References 21

Supplementary Methods. Exome sequencing and analysis, Immunohistochemistry and Western blotting.

Exome sequencing and analysis: Whole exome sequencing was performed by first fragmenting DNA and then creating libraries that were enriching for exon-coding regions using various capture reagents. The capture reagent varied depending on where and when the capture was performed as exome sequencing was completed at the Baylor College of Medicine, the University College London and the University of Manchester (details on each sample are given in Supplementary Table 1). The capture reagent was either Illumina's TruSeq capture reagent (Illumina Inc., San Diego, CA), Agilent's SureSelect capture reagent (Agilent Technologies, Santa Clara, CA), or Roche Nimblegen's Baylor VCRome capture reagent (Roche NimbleGen, Madison, WI). Capture was performed according to the manufacturer's protocol. Next-generation sequencing was performed on Illumina HiSeq 2000 (Illumina, San Diego, CA) for all samples. Sequence reads were aligned to the hg19 iteration of the reference human genome using BWA (v 0.5.9)[1]. Base score recalibration and local realignment for indel (insertion or deletion) detection and duplicate removal[2,3] were performed with GATK. SNVs were called using Samtools mpileup (version 0.1.17)[4] and short indels (insertions and deletion) were called using Samtools[5], Atlas-INDEL[6], and GATK[3] (indels included in our variant list had to be detected by all three programs, to decrease false-positive rates). Variants were annotated with ANNOVAR[7]. Protein-impacting variants that are rare (minor allele frequency <1%) or novel were preferentially explored. Candidate genes and variants were then assessed using databases such as dbNSFP which annotates the functional impact and the conservation of the mutated residues[8], Uniprot for the function of the proteins[9], NeXtProt[10] for the expression pattern, Mouse Genome Informatics[11] and NCBI's OMIM[12] for the phenotypes in mice and humans, and finally Genedistiller2[13] for a combination of some of the above databases. Homozygosity mapping from exome data using VCF variant files was achieved using HomozygosityMapper[14] and regions of homozygosity were analyzed for known disease-causing genes using the Genomic Oligoarray and SNP array evaluation tool v1.0[15]. We define variants as rare when they have a minor allele frequency (MAF) below 1% in the Exome Variant Server[16], as novel when absent from this database, and as protein-impacting when variants change the coding sequence of the protein (substituting an amino acid, causing an in-frame or out-of-frame deletion or insertion, a premature stop codon or altering a stop codon) or potentially affect splicing (nucleotide change within five bases of splice-donor or splice-acceptor sites).

Immunohistochemistry: C57BL/6 whole embryos (E16.5) or limbs (P2) were fixed in 4% PFA at 4°C overnight, washed with PBS, dehydrated using ethanol then embedded in paraffin for sectioning. Sectioned tissues were deparaffinized with xylene then rehydrated. Sections were rinsed with PBS, blocked with

3% Donkey Serum in 0.1% BSA and 0.1% Triton. Slides were then incubated overnight with a mouse monoclonal IgG_1 antibody against an epitope mapping between amino acids 437-559 at the C-terminus of TBC1D24 of human origin (clone G-6, catalog number sc-390237, Santa Cruz Biotechnology, Santa Cruz, CA). The antibody is provided at 200 µg/ml and was diluted at 1:200 for the immunohistochemistry (a control slide without primary antibody was also included). After rinsing, the slides were incubated for 1h in Alexa Fluor® 594 goat anti-rabbit IgG (Invitrogen product number A-11012, 1:600 final dilution in blocking buffer). After further rinsing, slides were mounted with ProLong® Gold antifade reagent with DAPI for imaging. Slides were viewed with a Zeiss Axioplan 2 fluorescence microscope, and images were taken using the same exposure parameters consistently for all images.

Western blotting: Proteins were extracted in 50 mM Tris-HCl pH7.5, 150 mM NaCl, 1% Triton X100, 1X Complete Protease Inhibitor Cocktail (Roche NimbleGen, Madison, WI), resolved in 4-15% gradient SDS-PAGE gels (Bio-Rad, Hercules, CA) and transferred to PVDF membranes for western blot analyses. For TBC1D24, the same antibody was used as for immunohistochemistry, at a dilution of 1:1000 (for the mouse brain lysate) or 1:100 (for the human fibroblast lysate), then an HRP-conjugated rabbit anti-mouse IgG antibody was used for detection (1:10,000 dilution, BioRad, Hercules, CA), and for control, a mouse monoclonal antibody to GAPDH directly conjugated to HRP (1:20,000 dilution, Clone GAPDH-71.1, Sigma-Aldrich Co, St-Louis, MO) was used.

Supplementary Table 1. Genetic analyses performed for each affected individual.

Individual number	Exome sequencing	Genetic mapping	Sanger sequencing
1	Exome sequencing (Nimblegen Baylor VCRome, 90X average coverage, 90% targeted base covered at ≥20X).		Sanger sequencing confirmation of *TBC1D24* mutations in proband and parents.
2a	Exome sequencing (Nimblegen Baylor VCRome, 95X average coverage, 90% targeted base covered at ≥20X).		Sanger sequencing confirmation of *TBC1D24* mutations in proband and parents
2b			Sanger sequencing for *TBC1D24* mutations identified in sibling. Confirmation of mutations in each parent.
3	Exome sequencing (Nimblegen Baylor VCRome, 144X average coverage, 95% targeted base covered at ≥20X).		Sanger sequencing confirmation of *TBC1D24* mutations in proband and parents.
4	Exome sequencing (Nimblegen Baylor VCRome, 72X average coverage, 86% targeted base covered at ≥20X).	Homozygosity mapping by SNP array.	Sanger sequencing confirmation of *TBC1D24* mutations in proband and parents.
5a	Exome sequencing, completed after the initial 15 sample analysis (Nimblegen Baylor VCRome, 99X average coverage, 92% targeted base covered at ≥20X).		Sanger sequencing confirmation of *TBC1D24* mutations in proband and parents.
5b			Sanger sequencing for variants identified in sibling. Sanger sequencing confirmation of *TBC1D24* mutations in proband and parents.
6			Sanger sequencing for *TBC1D24*. Confirmation of *TBC1D24* mutations in one parent.
7			Sanger sequencing for *TBC1D24*. Confirmation of *TBC1D24* mutations in parents.
8	Exome sequencing (Illumina TruSeq, 70X average coverage, 89% targeted base covered at ≥20X).	Homozygosity mapping from exome data.	Sanger sequencing confirmation of *TBC1D24* mutations in proband and parents.
9	Exome sequencing (Agilent SureSelect, 105X average coverage, 92% targeted base covered at ≥20X).		Sanger sequencing confirmation of *TBC1D24* mutations in proband and parents.
10			Sanger sequencing for *TBC1D24.*
11	Exome sequencing (Nimblegen Baylor VCRome, 122X average coverage, 91% targeted base covered at ≥20X).		
12a	Exome sequencing (Nimblegen Baylor VCRome, 94X average coverage, 90% targeted base covered at ≥20X).	Haplotype mapping by SNP array.	
12b		Haplotype mapping by SNP array.	
13			Sanger sequencing for *TBC1D24.*

14			Sanger sequencing for *TBC1D24.*
15	Exome sequencing (Nimblegen Baylor VCRome, 90X average coverage, 90% targeted base covered at ≥20X).		
16	Exome sequencing (Agilent SureSelect, 59X average coverage, 74% targeted base covered at ≥20X).		
17			Sanger sequencing for *TBC1D24.*
18	Exome sequencing, completed after the initial 15 sample analysis (Nimblegen Baylor VCRome, 135X average coverage, 90% targeted base covered at ≥20X).		Homozygosity mapping from exome data.
19	Exome sequencing (Nimblegen Baylor VCRome, 171X average coverage, 95% targeted base covered at ≥20X).		
20			Sanger sequencing for *TBC1D24.*
21			Sanger sequencing for *TBC1D24.*
22	Exome sequencing (Agilent SureSelect, 54X average coverage, 73% targeted base covered at ≥20X).		
23	Exome sequencing (Agilent SureSelect, 57X average coverage, 72% targeted base covered at ≥20X).	Homozygosity mapping from exome data.	
24	Exome sequencing (Nimblegen Baylor VCRome, 125X average coverage, 94% targeted base covered at ≥20X).	Homozygosity mapping by SNP array.	
25			Sanger sequencing for *TBC1D24.*
26	Exome sequencing (Illumina TruSeq, 33X average coverage, 66% targeted base covered at ≥20X).		

Supplementary Table 2. Primers used for Sanger sequencing and real-time PCR.

Primer name	Oligonucleotide sequence
**Sanger sequencing of human genomic DNA (coding exons and intron-exon boundaries for *TBC1D24* variant 1, NCBI CCDS ID#55980.1)**	
TBC1D24ex2F	TTTAGCCACTCTGTCCTCCC
TBC1D24ex2R	TCACGCCAGACACGTCC
TBC1D24ex3F	GGGGATCGGTACTCACACTAAC
TBC1D24ex3R	AGTCAGCCTGGTGGAAAGAC
TBC1D24ex4F	GCTCTGGGGCATACCTCG
TBC1D24ex4R	TCTGTGGGCAGGACACG
TBC1D24ex5F	GAGGGTGTGCAGGGTGAC
TBC1D24ex5R	GAAGCCCATCAGAGCCAG
TBC1D24ex6F	TAGTCTGGAGCACAGGGACG
TBC1D24ex6R	GGTGCTCCTGGAGGGATG
TBC1D24ex7F	ATGAAACGGGTTGTGGCTC
TBC1D24ex7R	CTTCAGCTGCCCGGACC
TBC1D24ex8F2	GCCTGGGTCAGTGCTGATAG
TBC1D24ex8R2	GGCTGCCTAGAGAGGCTCAG
**Real-time PCR for human samples**	
hTBC1D24-1:1255U28	TTTGGGACCGGAGAATGCTTTGTGTTTA
hTBC1D24-1:1441L26	TCGGTCTTGGAGGGCAGGTTGAAGTG
**Real-time PCR for mouse samples**	
mTbc1d24-1:5563U30	AGACCTGCTCTCTCATATCTTCACTAAATC
mTbc1d24-1:5714L26	TGGCACTCATGCTTGACATAACAACT

Supplementary Table 3. Table illustrating the process by which we identified recurrent mutations in TBC1D24 in the cohort of individuals with DOOR/S syndrome. As described in the methods and results, the automated variant identification pipeline leaves several “false-positive” variants which need to be visually assessed on the exome alignments and compared to controls.

Exomes with genes in common (every possible combination of exomes)	Number of genes in the automated output following an autosomal recessive inheritance pattern (1 homozygous variant or 2 heterozygous variants)	Number of genes excluded by visualization (exclusion of false-positive calls from the automated pipeline).	Genes remaining after visualization and curation of the candidate list
15 exomes	1	1	0
14 exomes	5	5	0
13 exomes	3	3	0
12 exomes	3	3	0
11 exomes	8	8	0
10 exomes	8	8	0
9 exomes	10	10	0
8 exomes	24	24	0
7 exomes	25	25	0
6 exomes	31	30	1 (*TBC1D24*)

Supplementary Table 4. Annotations for all known variants in *TBC1D24*.

Cohort	hg19 position	dbSNP ID	cDNA position (NM_001199107.1)	Protein variant	Variant type	Minor allele frequency (MAF) in EVS/number of chromosomes	Homozygosity frequency in EVS	Conservation Score PhastCons*	Conservation Score GERP*	Grantham Score*	Polyphen2 (Class:Score)*
DOORS syndrome	chr16:2546207C>G	rs201257588	c.58C>G	p.(Gln20Glu)	missense	0/12714	0	0.996	5.6	29	N/A
DOORS syndrome	chr16:2546267C>T	N/A	c.118C>T	p.(Arg40Cys)	missense	0/12620	0	0.959	5.6	180	probably-damaging:1
DOORS syndrome	chr16:2546268G>T	N/A	c.119G>T	p.(Arg40Leu)	missense	0/12634	0	0.995	5.6	102	probably-damaging:1
DOORS syndrome	chr16:2546477G>A	N/A	c.328G>A	p.(Gly110Ser)	missense	0/12768	0	0.831	5.6	56	probably-damaging:1
DOORS syndrome	chr16:2546873C>T	N/A	c.724C>T	p.(Arg242Cys)	missense	0/12732	0	0.996	4.2	180	probably-damaging:1
DOORS syndrome	chr16:2548254G>T	N/A	c.999G>T	p.(Leu333Phe)	missense	0/12722	0	0.997	2.55	22	probably-damaging:1
DOORS syndrome	chr16:2548263delT	N/A	c.1008delT	p.(His336Glnfs*12)	frameshift	2/12234=0.00033	0	0.216	5.6	N/A	N/A
DOORS syndrome	chr16:2549426G>A	N/A	c.1206+5G>A	splicing	intron	0/12432	0	0.97	4.71	N/A	N/A
Other epileptic syndromes	chr16:2546588G>C	N/A	c.439G>C	p.(Asp147His)	missense	1/12827=0.0001	0	0.476	5.6	81	probably-damaging:1
Other epileptic syndromes	chr16:2546617C>A	N/A	c.468C>A	p.(Cys156*)	stop-gain	0/12896	0	1	4.11	N/A	N/A
Other epileptic syndromes	chr16:2546835T>C	N/A	c.686T>C	p.(Phe229Ser)	missense	0/12672	0	0.877	5.43	155	probably-damaging:0.999
Other epileptic syndromes	chr16:2546900T>C	N/A	c.751T>C	p.(Phe251Leu)	missense	0/12738	0	1	5.24	22	possibly-damaging:0.871
Other epileptic syndromes	chr16:2547714_2547715delGT	N/A	c.969_970delGT	p.(Ser324Thrfs*3)	frameshift	N/A	0	0.991	-4.68	N/A	N/A
Other epileptic syndromes	chr16:2550823C>T	N/A	c.1544C>T	p.(Ala515Val)	missense	0/12790	0	0.965	5.79	64	probably-damaging:1
EVS	chr16:2546171T>C	rs77585883	c.22T>C	p.(Cys8Arg)	missense	1/12653=0.00016	0	1	4.48	180	benign:0.0
EVS	chr16:2546225G>T	N/A	c.76G>T	p.(Glu26*)	stop-gain	1/12713=0.00016	0	1	4.63	N/A	N/A
EVS	chr16:2546226A>T	N/A	c.77A>T	p.(Glu26Val)	missense	1/12711=0.00016	0	1	5.6	121	benign:0.007
EVS	chr16:2546318C>T	rs202162520	c.169C>T	p.(Arg57Cys)	missense	25/12695=0.00393	0	1	4.59	180	probably-damaging:0.99

											4
EVS	chr16:2546319G>A	N/A	c.170G>A	p.(Arg57His)	missense	1/12779=0.00016	0	0.997	2.59	29	benign:0.002
EVS	chr16:2546327C>T	N/A	c.178C>T	p.(Arg60Trp)	missense	1/12765=0.00016	0	0.935	3.48	101	probably-damaging:0.994
EVS	chr16:2546328G>A	rs200226466	c.179G>A	p.(Arg60Gln)	missense	5/12767=0.00078	0	0.629	-0.34	43	benign:0.03
EVS	chr16:2546346C>T	N/A	c.197C>T	p.(Thr66Met)	missense	1/12821=0.00016	0	0.953	5.6	81	possibly-damaging:0.645
EVS	chr16:2546366G>A	N/A	c.217G>A	p.(Val73Met)	missense	2/12856=0.00031	0	1	5.6	21	probably-damaging:1.0
EVS	chr16:2546489G>A	N/A	c.340G>A	p.(Val114Met)	missense	2/12780=0.00031	0	0.998	5.6	21	probably-damaging:1.0
EVS	chr16:2546492C>T	N/A	c.343C>T	p.(Arg115Cys)	missense	1/12779=0.00016	0	1	5.6	180	probably-damaging:1.0
EVS	chr16:2546493G>A	rs201174513	c.344G>A	p.(Arg115His)	missense	1/12785=0.00016	0	1	4.63	29	benign:0.1
EVS	chr16:2546606G>A	N/A	c.457G>A	p.(Glu153Lys)	missense	1/12873=0.00016	0	0.997	5.27	56	probably-damaging:1.0
EVS	chr16:2546642G>A	rs200926225	c.493G>A	p.(Gly165Ser)	missense	22/12798=0.00343	0	0.072	-1.79	56	benign:0.001
EVS	chr16:2546790G>A	rs200324356	c.641G>A	p.(Arg214His)	missense	14/12708=0.0022	0	1	5.43	29	probably-damaging:0.997
EVS	chr16:2546840G>A	N/A	c.691G>A	p.(Val231Ile)	missense	1/12699=0.00016	0	0.959	5.43	29	possibly-damaging:0.935
EVS	chr16:2546880C>T	N/A	c.731C>T	p.(Ala244Val)	missense	1/12727=0.00016	0	0.153	5.24	64	probably-damaging:1.0
EVS	chr16:2546883T>C	N/A	c.734T>C	p.(Leu245Pro)	missense	1/12725=0.00016	0	0.927	5.24	98	probably-damaging:1.0
EVS	chr16:2546934C>T	rs201060500	c.785C>T	p.(Ser262Leu)	missense	32/12720=0.00502	0	0.467	5.24	145	possibly-damaging:0.934
EVS	chr16:2546957C>T	N/A	c.808C>T	p.(Arg270Cys)	missense	1/12851=0.00016	0	0.994	5.24	180	probably-damaging:1.0
EVS	chr16:2547020G>A	N/A	c.871G>A	p.(Ala291Thr)	missense	1/12827=0.00016	0	0.959	4.09	58	possibly-damaging:0.863
EVS	chr16:2547026C>T	N/A	c.877C>T	p.(Arg293Cys)	missense	1/12835=0.00016	0	1	5.09	180	probably-damaging:1.0
EVS	chr16:2547027G>A	rs199700840	c.878G>A	p.(Arg293His)	missense	7/12807=0.00109	0	1	5.09	29	probably-damaging:1.0
EVS	chr16:2547034C>G	rs72768728	c.885C>G	p.(Phe295Leu)	missense	106/12472=0.01685	0.00016	0.989	-0.32	22	benign:0.114
EVS	chr16:2547101G>A	N/A	c.952G>A	p.(Val318Met)	missense	1/12473=0.00016	0	0.94	5.09	21	probably-damaging:0.99

											2
EVS	chr16:2547122G>T	N/A	c.965+8G>T	Near splice site	Intronic	1/12371=0.00016	0	0	-4.14	N/A	N/A
EVS	chr16:2548294G>A	N/A	c.1039G>A	p.(Val347Met)	missense	1/12751=0.00016	0	1	5.6	21	probably-damaging:0.999
EVS	chr16:2548307G>A	N/A	c.1052G>A	p.(Arg351Lys)	missense	1/12769=0.00016	0	0.995	5.6	26	probably-damaging:0.993
EVS	chr16:2548327C>A	N/A	c.1072C>A	p.(Pro358Thr)	missense	1/12651=0.00016	0	1	5.6	38	probably-damaging:1.0
EVS	chr16:2549352C>T	rs73490287	c.1143-6C>T	Near splice site	intronic	242/12140=0.03909	0.00113	0.179	-0.84	N/A	N/A
EVS	chr16:2549411C>T	rs61731477	c.1196C>T	p.(Thr399Met)	missense	35/12471=0.0056	0	0.991	5.67	81	possibly-damaging:0.94
EVS	chr16:2549891A>C	N/A	c.1262A>C	p.(Lys421Thr)	missense	1/12319=0.00016	0	0.995	5.65	78	benign:0.328
EVS	chr16:2550293G>A	rs141399869	c.1327G>A	p.(Glu443Lys)	missense	22/12426=0.00353	0	0.992	5.36	56	probably-damaging:0.993
EVS	chr16:2550333C>T	rs200641000	c.1367C>T	p.(Pro456Leu)	missense	2/12572=0.00032	0	0	2.01	98	benign:0.0
EVS	chr16:2550347G>A	rs201911646	c.1381G>A	p.(Ala461Thr)	missense	2/12600=0.00032	0	0	-3.57	58	benign:0.0
EVS	chr16:2550350G>A	N/A	c.1384G>A	p.(Glu462Lys)	missense	2/12606=0.00032	0	0.681	4.2	56	benign:0.0
EVS	chr16:2550377G>A	N/A	c.1411G>A	p.(Ala471Thr)	missense	1/12561=0.00016	0	0	-10.2	58	benign:0.0
EVS	chr16:2550393C>A	rs202216463	c.1427C>A	p.(Ala476Asp)	missense	38/12436=0.00609	0	0.003	3.84	126	possibly-damaging:0.835
EVS	chr16:2550456T>C	N/A	c.1490T>C	p.(Met497Thr)	missense	1/12395=0.00016	0	1	5.49	81	possibly-damaging:0.848
EVS	chr16:2550849C>T	rs78644690	c.1570C>T	p.(Arg524Trp)	missense	1/12765=0.00016	0	1	5.79	101	benign:0.106
EVS	chr16:2550904A>G	N/A	c.1625A>G	p.(Asn542Ser)	missense	1/12767=0.00016	0	1	3.39	46	benign:0.001
EVS	chr16:2550921G>A	rs201649140	c.1642G>A	p.(Val548Met)	missense	2/12718=0.00031	0	0.039	-2.49	21	benign:0.115

*Notes: N/A, Not available. Data for the mutations in DOORS and other epileptic syndromes were generated using the SeattleSeq Annotation server: http://snp.gs.washington.edu/SeattleSeqAnnotation137. Data for other *TBC1D24* variants found in the population are from the Exome Variant Server (EVS), NHLBI GO Exome Sequencing Project, Seattle, WA (http://evs.gs.washington.edu/EVS/), data release ESP6500SI-V2 accessed July 27th 2013. PhastCons score[17]: conservation of the mutated nucleotide among 17 vertebrate species, with 1 being the most conserved. Genomic Evolutionary Rate Profiling (GERP) score[18]: A score for constrained DNA elements in 29 mammalian species, ranges from -12.3 to 6.17, with 6.17 being the most conserved. Grantham score[19]: Categorizes codon replacements into classes of increasing chemical dissimilarity, ranges from 5 to 215, with 5 being the most similar.PolyPhen2 (Class:Score): Prediction of possible impact of an amino acid substitution on protein structure and function based on Polymorphism Phenotyping (PolyPhen2) program. It lists both the PolyPhen2 prediction class and the score separated by a colon.

Supplementary Table 5. Candidate regions from SNP array or homozygosity mapping of exome data.

**Individual 4. Homozygous for *TBC1D24* mutation. Region containing *TBC1D24* is emboldened below.**	**Individual 8. Homozygous for *TBC1D24* mutation. Region containing *TBC1D24* is emboldened below.**	**Individuals 12a and b. *TBC1D24* is not mutated and is not in the regions of haplotype sharing between affected siblings.**	**Individual 18. *TBC1D24* is not mutated and is not in the regions of homozygosity.**	**Individual 23. *TBC1D24* is not mutated and is not in the regions of homozygosity.**	**Individual 24. *TBC1D24* is not mutated and is not in the regions of homozygosity.**
chr1:18559122-48222050	chr2:905687-1926437	chr1:8828888-18505944	chr1:19166294-19549343	chr1:12779560-14143003	chr1:35369014-36652619
chr1:73064758-74147725	chr3:187416666-197566254	chr1:36909349-38597706	chr1:34383662-36814473	chr1:65113568-84880380	chr1:49352177-50648032
chr1:142535628-149671593	chr4:175598334-185587165	chr2:43239638-49496567	chr1:43675467-44569098	chr1:92647532-94343233	chr2:62859647-64434512
chr1:169248783-181528472	chr4:187179210-189018486	chr2:75860115-159321442	chr1:49332969-53333116	chr1:156526444-156640678	chr2:223436607-237629101
chr1:216813244-235760817	chr7:142626549-148851213	chr3:170726718-188139229	chr1:92262874-94335194	chr1:158390252-158655036	chr3:48964772-50431592
chr2:18674-1531755	chr8:8234077-17612875	chr4:1-6171166	chr1:151109641-151413613	chr2:11738091-74007136	chr3:163500280-164906038
chr2:15106804-20381830	chr8:19221700-24811065	chr4:35994365-39863991	chr1:152278924-152538358	chr2:168099738-168115769	chr4:102076982-103228545
chr2:81191209-82548471	chr8:29927300-38854041	chr4:72851507-83647447	chr1:153747616-154245142	chr2:186655726-189875421	chr6:74517352-75539142
chr2:95344824-98673494	chr8:144732418-144809804	chr5:63147642-76611767	chr1:155059851-156107532	chr2:207621759-209224875	chr6:145518707-146575337
chr3:30200658-46704229	chr10:128202435-129917560	chr5:94735770-107223054	chr1:216595306-220928313	chr2:219903258-220333950	chr7:68908066-69952187
chr3:48413537-49708502	chr10:135076596-135184126	chr6:27102585-28363728	chr1:227968222-228482010	chr3:5241309-13421150	chr8:2275178-4122628
chr3:88636898-89960347	chr11:237087-400109	chr8:101942255-127935307	chr2:20884550-30381505	chr3:46399798-47539751	chr8:4559317-5745690
chr3:157313589-172002955	chr11:5172786-5373646	chr10:30691954-77076960	chr2:45774050-56603118	chr3:182631792-184429414	chr8:6160312-8433876
chr3:180368253-181375474	chr15:51783820-52689631	chr12:27552995-96409294	chr2:212242745-216973890	chr4:103911069-126373789	chr8:47768915-48852225
chr4:427376-1603196	chr15:89169858-89402596	chr12:125502505-126740922	chr3:48019258-48897078	chr4:167012381-177073026	chr8:112021630-113403001
chr4:2403365-6550849	chr16:103517-1447278	chr13:1-21704372	chr3:49734377-51752020	chr5:52942083-55155402	chr9:41626142-47212247
chr4:146875551-183569127	**chr16:1877698-2891212**	chr14:52533961-56368086	chr3:51907736-52467551	chr9:125239253-127101924	chr9:65529441-68167836
chr5:27308663-46181048	chr16:3170188-3724465	chr14:85900219-101102993	chr3:174814920-182631654	chr10:123298158-125506302	chr10:55418087-59905060
chr5:50237267-69840276	chr17:43342141-44248814	chr14:102590515-106353025	chr4:78106123-88536901	chr10:134997480-135233541	chr10:83600968-95398330
chr5:70392462-73380747	chr17:67031457-70943990	chr15:1-34007269	chr4:88536952-98965696	chr11:5373646-5510497	chr11:48735952-50056195
chr6:27741682-28982287	chr19:15508362-15760881	chr15:38349182-55907488	chr4:166159933-175899001	chr11:56756399-58478084	chr11:65642495-118743286
chr6:83619375-137573291	chr19:37441111-37488499	chr15:64289669-89823662	chr5:138876953-140052424	chr11:66099987-68549340	chr12:88324226-89339129
chr7:18630208-89807586	chr22:29704662-44681612	chr16:53562822-77713252	chr5:140626571-141014494	chr11:119005088-120175749	chr16:32139546-33828679
chr8:654796-19867220		chr17:1729707-75689041	chr5:159912418-167674370	chr12:10958658-11339020	chr17:27916023-29025184
chr8:120216957-128330178		chr20:32270552-32928371	chr6:42146663-43753212	chr14:92084004-97321689	chr22:28235162-29457582
chr9:47352-3765395		chr21:22104937-26771217	chr6:151365956-165693624	chr16:20638576-27356203	
chr9:43382804-46433829			chr7:12644033-23353160	chr16:71318001-72832135	
chr9:65529441-70984188			chr7:23353231-31127158	chr17:17075181-20163529	
chr9:138482453-141114095			chr9:8500690-27047241	chr17:26824156-36493598	
chr10:5624038-26020424			chr9:30690073-33289187	chr17:39646021-40048613	
chr10:46208186-47587136			chr9:33799218-34724987	chr19:44500478-44610798	
chr10:108667108-120109482			chr9:34725069-39149855	chr19:52869022-53077411	
chr11:95724719-106523224			chr9:70871944-90733797		
chr12:1618202-3183027			chr10:5324876-11047307		
chr12:10131684-11226421			chr10:74268031-76854564		
chr12:79995615-87922782			chr10:116335246-125780758		
chr13:20221855-25110139			chr10:125780759-131334768		
chr14:38592912-39685539			chr11:6007772-6585007		
chr15:25092266-36594061			chr11:65617324-66262606		

chr15:64675313-67467507<br>chr15:82451052-83768391<br>**chr16:61971-5863931**<br>chr16:31857841-34277622<br>chr17:4256652-13681058<br>chr17:67449386-75661063<br>chr18:10313183-11510046<br>chr18:12072364-15410816<br>chr18:18546888-29235471<br>chr19:1512093-8358349<br>chr20:33451706-34853295<br>chr21:27658905-32218481<br>chr22:31532960-32569263<br>chr22:42867332-51243435			chr12:88627488-106410149<br>chr12:106410273-129298779<br>chr15:32744836-40582167<br>chr15:40582169-45361180<br>chr15:45365905-50784950<br>chr15:50785054-63349096<br>chr16:20410547-21145648<br>chr16:46435519-49764948<br>chr16:66861966-67679348<br>chr17:7286326-7664239<br>chr17:16748833-18314913<br>chr17:19091317-20321413<br>chr17:27905673-29848460<br>chr17:37790371-38609187<br>chr17:39967442-40459562<br>chr17:40818584-41196408<br>chr18:26245796-29709473<br>chr18:29709613-36915294<br>chr19:36633277-38688977<br>chr19:42474200-43285368<br>chr22:23242127-24579049<br>chr22:25574507-29104955<br>chr22:29806132-33670584		

Supplementary Table 6. Review of the expression data on *TBC1D24* in human and mouse tissues, detected at the level of mRNA or protein, from publications or public databases. See also Supplementary Figure 1.

Source and reference	Method	Species	Summary	Link
Manuscript by Guven *et al.*[20]	RNA - real-time PCR	Mouse	High expression of the RNA in the parietal cortex, corpus callosum and brainstem, and they showed that non-neural tissues express isoform 2 that lacks the short third exon.	
Manuscript by Falace *et al.*[21]	RNA *in situ* hybridization	Mouse	High expression of the RNA in the brain, more specifically in the deep cortical layers of the neocortex and in the hippocampus.	
Allen Brain Atlas[22]	RNA *in situ* hybridization	Mouse	Highest levels are seen in the isocortex (all regions including auditory areas, highest in somatomotor area) and the hippocampal formation (Ammon's horn and the dentate gyrus).	http://mouse.brain-map.org/experiment/show?id=69531049.
Eurexpress Transcriptome Atlas[23]	RNA *in situ* hybridization	Mouse	High levels of RNA are detected in the embryonic mouse brain, spinal cord, peripheral nervous system, ganglia, eye, nose and liver.	http://www.eurexpress.org/ee/databases/assayMontage.jsp?assayID=euxassay_010949.
BioGPS[24]	RNA microarray	Mouse	Higher levels in the salivary and adrenal glands, various bone marrow-derived cells, osteoclasts, osteoblasts, retinal cells, and various regions of the brain (cortex, amygdala, hippocampus, hypothalamus, cerebellum, olfactory bulb).	http://biogps.org/#goto=genereport&id=57465.
Geneinvestigator[25]	RNA microarray	Human and mouse	Highest expression in mice is in the cortex and hippocampus, and intermediate levels in other regions of the brain, the retina, the peripheral nervous system, the intestinal tract, osteoclasts, the liver and some bone marrow-derived cells. In human tissues, highest levels are in pyramidal neurons, various regions of the brain, the kidney, the salivary glands, the adipose tissue, osteoblasts, the thyroid, the liver and mammary glandular cells.	https://www.genevestigator.com.
RNA-seq Atlas[26]	RNA-seq	Human	High levels in the hypothalamus and the kidney.	http://medicalgenomics.org/rna_seq_atlas.
Human Protein Atlas[27]	Immunohistochemistry	Human	High levels were detected in glandular cells of the digestive tract and the uterus, in renal tubular cells, and in chondrocytes.	http://www.proteinatlas.org/ENSG00000162065/normal.

Supplementary Table 7. Coverage data for candidate genes. Included are the five genes encoding proteins with TLDc domain (including *TBC1D24*) and for *RAB3GAP2*, in the 10 samples without *TBC1D24* mutations. For *TBC1D24*, regions with low coverage were visually assessed on BAM files and Sanger sequenced if not covered, in order not to miss a mutation. The average proportion of coding bases with at least 10X coverage for *TBC1D24* was 100% for the Nimblegen Baylor VCRome capture reagent, 94% for the Agilent SureSelect capture reagent, and 90% for the Illumina TruSeq capture reagent.

**Gene name**	**Transcript ID**	**Average coverage for coding bases**	**% of coding bases with ≥10X coverage**
TBC1D24	NM_001199107	66X	97.2%
NCOA7	NM_001122842	125X	99.9%
OXR1	NM_001198532	131X	99.3%
KIAA1609	NM_020947	89X	95.4%
C20orf118	NM_080628	75X	92.8%
RAB3GAP2	NM_012414	147X	99%

Supplementary Table 8. Candidate gene analysis from exome data in 10 individuals without *TBC1D24* mutations. The same strategy for exome analysis was applied as described in the methods and Table 1. Genes with variants in three or fewer samples from the automated output are still being considered as candidates at the moment. The names of the genes where rare/novel variants were detected in multiple samples by the automated output for the recessive model are given in the next table.

	**Recessive inheritance model**		***De novo* dominant inheritance model**	
**Exomes with genes in common (every possible combination of cases)**	**Automated output: Genes with rare or novel variants**	**After visualization and removal of false-positives**	**Automated output: Genes with rare or novel variants**	**After visualization and removal of false-positives**
10 exomes	7	0	8	0
9 exomes	3	0	8	0
8 exomes	2	0	7	0
7 exomes	6	0	16	0
6 exomes	4	0	27	0
5 exomes	5	0	38	0
4 exomes	2	0	74	0
3 exomes	14	Analysis ongoing, candidate genes need consideration	165	Analysis ongoing, candidate genes need consideration
2 exomes	41		503	
Single exome	288		2604	

Supplementary Table 9. Gene names for recessive model from exome data in 10 individuals without *TBC1D24* mutations.

Samples with genes in common	Automated output: Genes with rare or novel variants
10	*MUC4, ZNF595, ZMAT1, ATP7A, VBP1, abParts, C21orf62*
9	*DGKK, ZNF761, BCORL1*
8	*TEX13A, ABP1*
7	*ARSD, HLA-B, VCX2, DNHD1, C17orf100, UHRF1*
6	*IQSEC1, TREH, MCF2, SEMA3B*
5	*LRRC37A, ZNF492, CCDC66, SGK110, NFKBIZ*
4	*HDHD1, FLJ22184*
3	*GGT2, MXRA5, GOLGA6L6, WWC3, WDR33, FAM86B2, FICD, ZNF705A, SOCS1, LOC728405, SYNM, SLC25A5, TMEM185A, MAL2*
2	*TRY6, CD177, CRIPAK, NBPF1, ZNF718, FAM75A3, ODZ1, SIRPA, AKR1D1, WWC1, TMEM191B, PCDH11Y, LILRB1, FAM90A10, MAGEA6, CXorf22, ZNF598, NACA, ADH5, C19orf55, BC073807, JPX, NBPF16, TRIL, CTAGE4, MAPK8IP2, AP1S3, AKAP3, RBBP7, LAMB2, DQ580909, OTC, AGAP5, C14orf169, MAGIX, AVPI1, TCEAL4, SERPINA7, OPN1LW, POLR2J2, LOC642846*

Supplementary Figure 1. Conservation across species of the residues affected by missense substitutions. The blue highlights the residues for the percentage of identity in this alignment (darker being the highest identity). PhastCons score[17]: conservation of the mutated nucleotide among 17 vertebrate species, with 1 being the most conserved. Genomic Evolutionary Rate Profiling (GERP) score[18]: a score for constrained DNA elements in 29 mammalian species, ranges from -12.3 to 6.17, with 6.17 being the most conserved. Grantham score[19]: categorizes codon replacements into classes of increasing chemical dissimilarity, ranges from 5 to 215, with 5 being the most similar. The alignment was performed using the Clustal Omega program[28] on the Uniprot website (www.uniprot.org). Graphical representation of the alignment was performed using JalView (www.jalview.org). Uniprot IDs for the proteins used in the alignment of TBC1D24 homologues are Q9ULP9 for Homo sapiens, F6TFP7 for the Rhesus macaque, F1Q1S9 for the dog, Q3UUG6 for the mouse, G3TG12 for the african elephant, G1DG11 for the goat, F1NEU9 for the chicken, Q08CX5 for the western clawed frog, F6S4P5 for the gray short-tailed opossum, E7FCR8 for the zebrafish, Q7Q0N9 for the fruit fly, D3DML7 for the african malaria mosquito, and H2KZ54 for the roundworm.

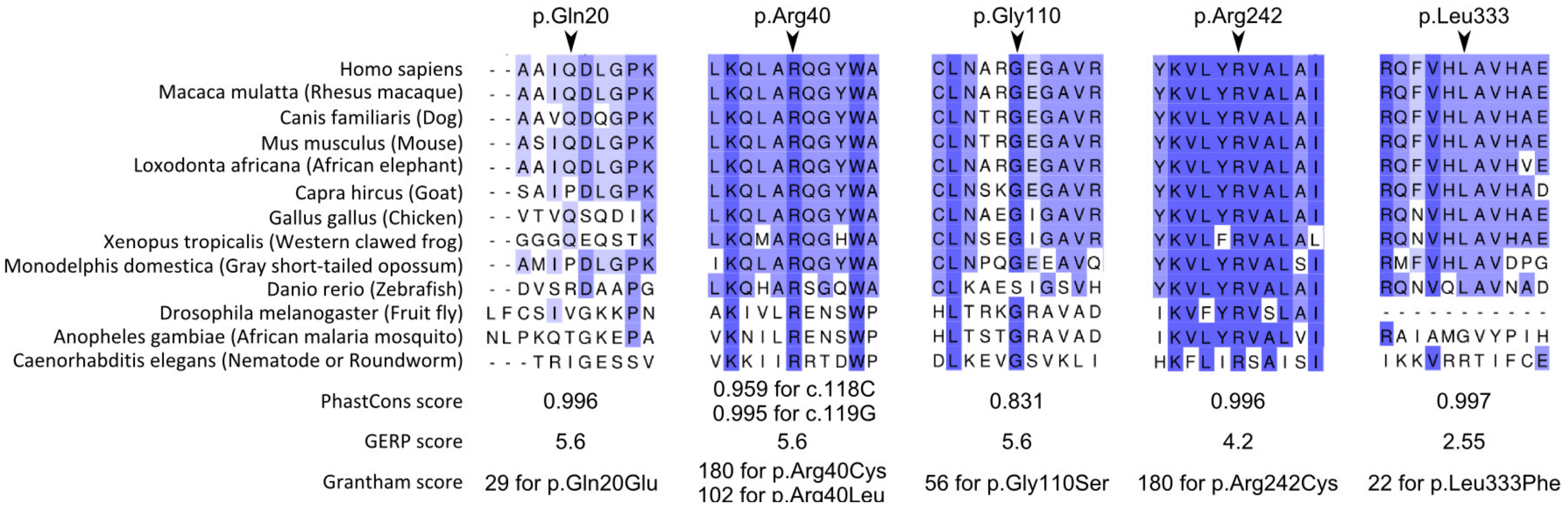

Supplementary Figure 2. Additional *Tbc1d24* expression data from public databases. A) Expression (blue) in adult mouse brain sagittal section using RNA ISH from the Allen Institute for Brain Science[22]. B) High expression (purple staining) in the embryonic mouse brain, spinal cord and liver from the Eurexpress Transcriptome Atlas[23]. C) High expression in the hypothalamus and kidneys by RNAseq from the RNA-seq Atlas[26].

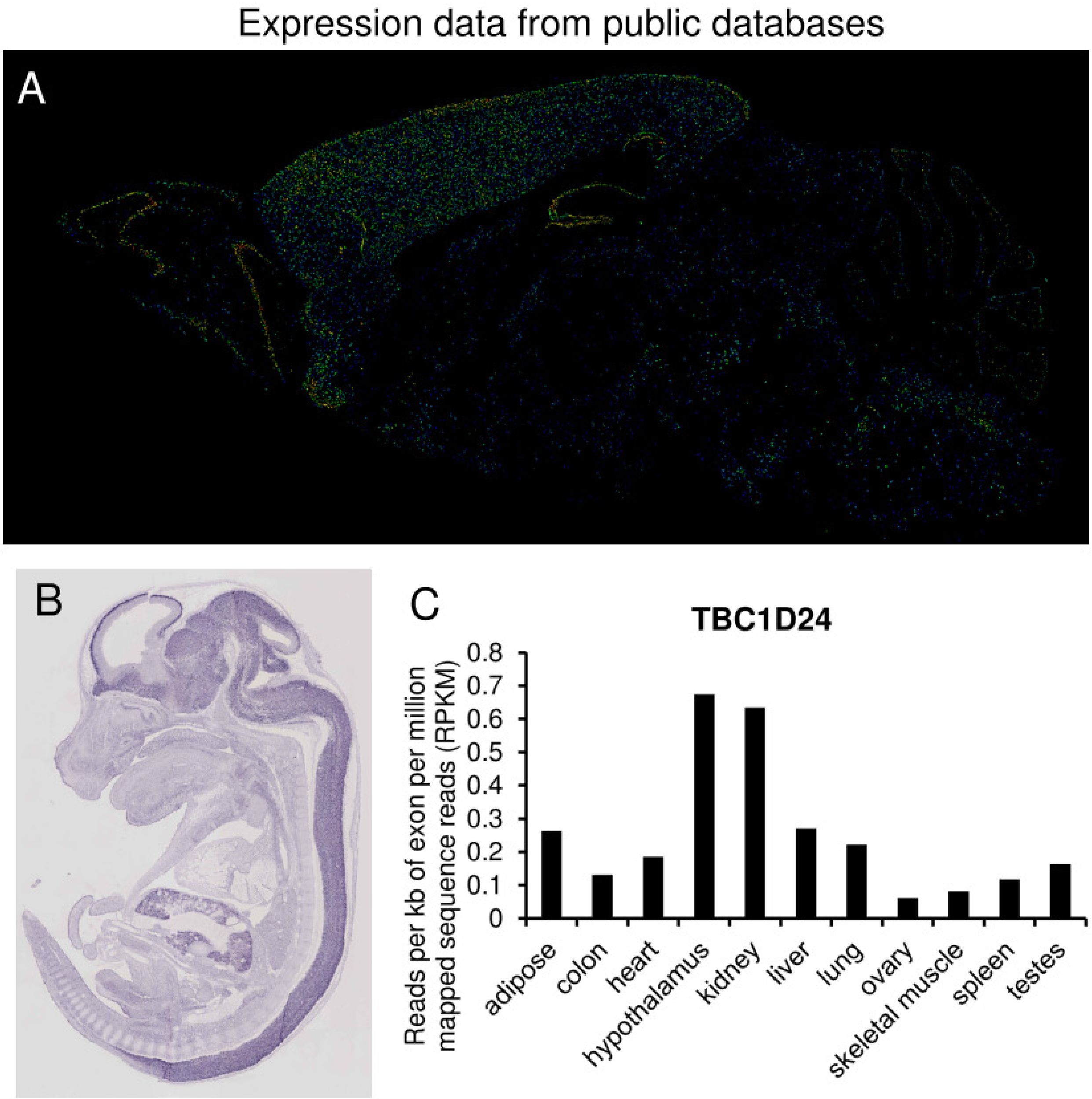

Supplementary Figure 3. Tbc1d24 expression profiling. A) Expression of Tbc1d24 in C57BL/6 mouse chondrocytes in the distal phalanges of the forelimbs at age P2. B) Same as in panel A but without the primary antibody to demonstrate the background signal. C) Detection of mouse Tbc1d24 in mouse embryonic brain at E16.5 (embryos selected to correlate with *in situ* hybridization data available in mouse embryos, see next figure). D) Control without primary antibody to demonstrate specificity of antibody. E) Western blot of mouse brain lysate protein to demonstrate the specificity of the antibody. The computed molecular weight of the unmodified isoform 1of mouse Tbc1d24 is 63 kDa. F) Expression analysis for *Tbc1d24* in various newborn mouse tissues An ANOVA on ranks analysis showed that the median expression varied between the tissues ($p=0.026$). *: $p<0.05$ by a comparison between the groups using Dunnett's method.

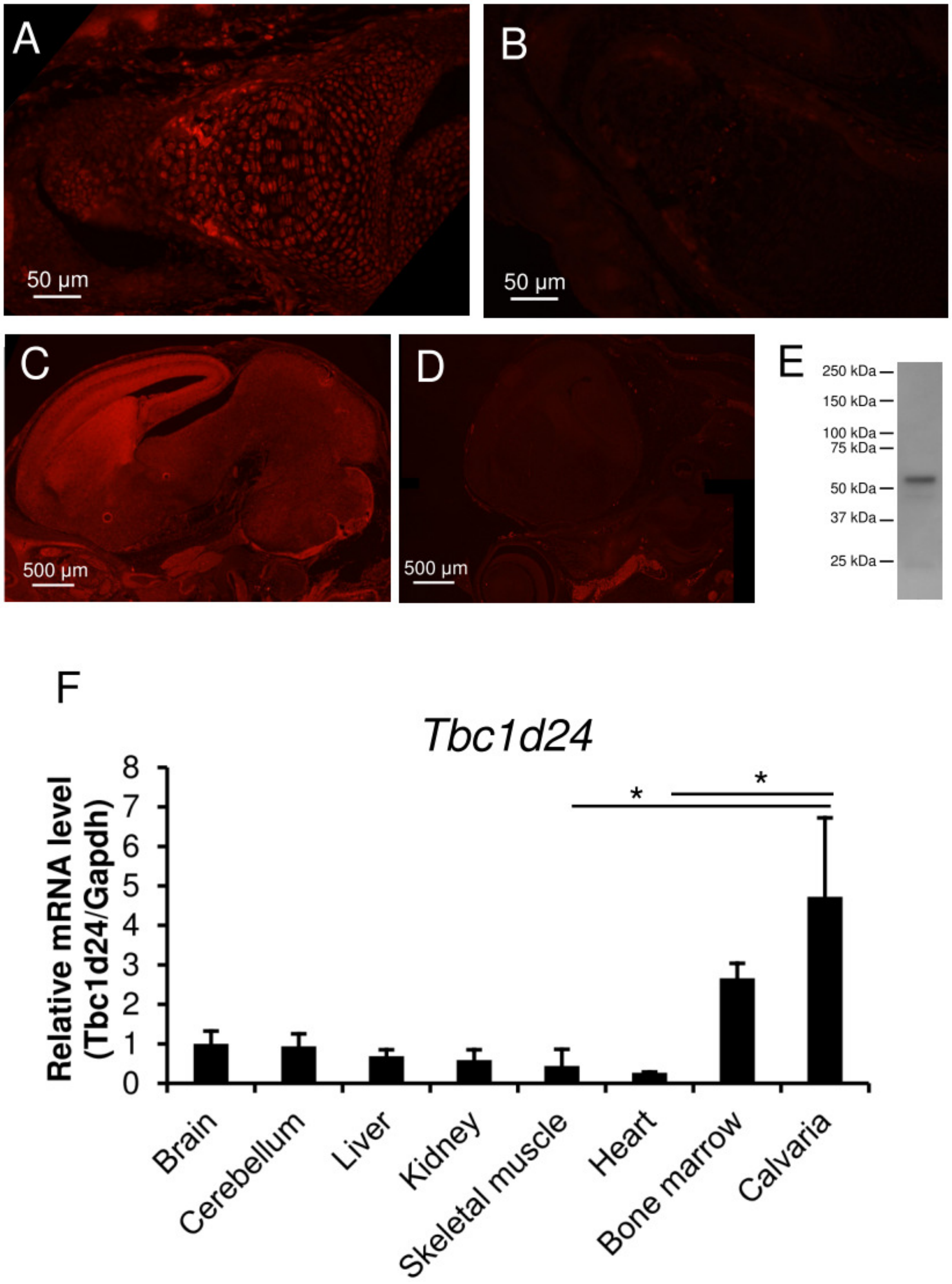

Supplementary Discussion. 2-Oxoglutaric aciduria and DOORS syndrome types.

Increased 2-oxoglutaric acid is often found in the blood and urine in DOORS syndrome, but is not pathognomonic. Levels can fluctuate between normal values to very high values over time in the same patient[29,30] and are not elevated in all affected individuals. As such, its absence cannot be used to exclude a diagnosis of DOORS syndrome. 2-oxoglutaric aciduria is found in several metabolic disorders, but rarely in association with dysmorphisms such as in DOORS syndrome[31]. Patton *et al.* noted that α-hydroxyglutarate, a metabolite of 2-oxoglutarate, is also elevated in the urine, thus suggesting that the activity of the 2-oxoglutarate dehydrogenase complex (made of the E1, E2 and E3 components) was intact[29]. Surrendran *et al.* found decreased E1 activity in patient fibroblasts and white blood cells[32], while it was normal in the patient described by James *et al.*[33] . Patton *et al.* suggested separating DOORS syndrome from autosomal dominant deafness and onychodystrophy[29], now called DDOD syndrome [MIM 124480], and James *et al.* also excluded from their review cases with either dominant inheritance or without intellectual disability or seizures[33]. Rajab *et al.* suggested dividing DOORS syndrome into type I, with more severe neurological involvement (in terms of intellectual disability and seizures) and type II with a milder neurological disease and course[34]. They noted that 2-oxoglutaric aciduria was present in the more severe cases and in none of the milder cases. However, as Felix *et al.* demonstrated, several individuals with type I (or more severe) DOORS syndrome do not have 2-oxoglutaric aciduria[35]. James *et al.* also concluded that the division between type I and type II should not be used, in part because of clinical heterogeneity even within families[33].

We suggest three hypotheses for the source of 2-oxoglutaric aciduria found in DOORS in light of our findings. 2-oxoglutarate might originate from increased glutamate release from neurons (suggested by increased neurotransmitter release in the Skywalker Drosophila studies[36]) with subsequent metabolism to 2-oxoglutarate in astrocytes. Another hypothesis is that vesicle-bound aspartate aminotransferase, converting 2-oxoglutarate and aspartate to glutamate for vesicular transport[37], could be secondarily affected by abnormal vesicular transport. We could partly test these hypotheses in the future using cerebrospinal fluid or magnetic resonance spectroscopy of the affected individuals. Finally, 2-oxoglutarate could originate from defective activity of the TLDc domain, the substrates and catalytic activity of which are unknown, but could involve 2-oxoglutarate. This is less likely given that the mutations are often far from the TLDc domain (see Figure 3D).

References

1. Li H, Durbin R. Fast and accurate short read alignment with Burrows-Wheeler transform. *Bioinformatics* 2009; **25**: 1754-60.
2. DePristo MA, Banks E, Poplin R, et al. A framework for variation discovery and genotyping using next-generation DNA sequencing data. *Nat Genet* 2011; **43**(5): 491-8.
3. McKenna A, Hanna M, Banks E, et al. The Genome Analysis Toolkit: a MapReduce framework for analyzing next-generation DNA sequencing data. *Genome Res* 2010; **20**(9): 1297-303.
4. Li H, Handsaker B, Wysoker A, et al. The Sequence Alignment/Map format and SAMtools. *Bioinformatics* 2009; **25**: 2078-9.
5. Akyuz M, Cabuk H. Meteorological variations of PM2.5/PM10 concentrations and particle-associated polycyclic aromatic hydrocarbons in the atmospheric environment of Zonguldak, Turkey. *J Hazard Mater* 2009; **170**(1): 13-21.
6. Havlak P, Chen R, Durbin KJ, et al. The Atlas genome assembly system. *Genome Res* 2004; **14**(4): 721-32.
7. Wang K, Li M, Hakonarson H. ANNOVAR: functional annotation of genetic variants from high-throughput sequencing data. *Nucleic Acids Res* 2010; **38**(16): e164.
8. Liu X, Jian X, Boerwinkle E. dbNSFP v2.0: A Database of Human Non-synonymous SNVs and Their Functional Predictions and Annotations. *Hum Mutat* 2013.
9. Uniprot_Consortium. Reorganizing the protein space at the Universal Protein Resource (UniProt). *Nucleic Acids Res* 2012; **40**(Database issue): D71-5.
10. Gaudet P, Argoud-Puy G, Cusin I, et al. neXtProt: organizing protein knowledge in the context of human proteome projects. *J Proteome Res* 2013; **12**(1): 293-8.
11. Shaw DR. Searching the Mouse Genome Informatics (MGI) resources for information on mouse biology from genotype to phenotype. *Curr Protoc Bioinformatics* 2009; **Chapter 1**: Unit1 7.
12. Baxevanis AD. Searching Online Mendelian Inheritance in Man (OMIM) for information on genetic loci involved in human disease. *Curr Protoc Hum Genet* 2012; **Chapter 9**: Unit 9 13 1-0.
13. Seelow D, Schwarz JM, Schuelke M. GeneDistiller--distilling candidate genes from linkage intervals. *PLoS One* 2008; **3**(12): e3874.
14. Seelow D, Schuelke M. HomozygosityMapper2012--bridging the gap between homozygosity mapping and deep sequencing. *Nucleic Acids Res* 2012; **40**(Web Server issue): W516-20.
15. Wierenga KJ, Jiang Z, Yang AC, Mulvihill JJ, Tsinoremas NF. A clinical evaluation tool for SNP arrays, especially for autosomal recessive conditions in offspring of consanguineous parents. *Genet Med* 2013; **15**(5): 354-60.
16. EVS. Exome Variant Server, NHLBI GO Exome Sequencing Project (ESP), Seattle, WA. 2012. http://evs.gs.washington.edu/EVS/.
17. Felsenstein J, Churchill GA. A Hidden Markov Model approach to variation among sites in rate of evolution. *Mol Biol Evol* 1996; **13**(1): 93-104.
18. Cooper GM, Stone EA, Asimenos G, Green ED, Batzoglou S, Sidow A. Distribution and intensity of constraint in mammalian genomic sequence. *Genome Res* 2005; **15**(7): 901-13.
19. Grantham R. Amino acid difference formula to help explain protein evolution. *Science* 1974; **185**(4154): 862-4.
20. Guven A, Tolun A. TBC1D24 truncating mutation resulting in severe neurodegeneration. *J Med Genet* 2013; **50**(3): 199-202.
21. Falace A, Filipello F, La Padula V, et al. TBC1D24, an ARF6-interacting protein, is mutated in familial infantile myoclonic epilepsy. *Am J Hum Genet* 2010; **87**(3): 365-70.
22. Lein ES, Hawrylycz MJ, Ao N, et al. Genome-wide atlas of gene expression in the adult mouse brain. *Nature* 2007; **445**(7124): 168-76.
23. Diez-Roux G, Banfi S, Sultan M, et al. A high-resolution anatomical atlas of the transcriptome in the mouse embryo. *PLoS Biol* 2011; **9**(1): e1000582.

24. Wu C, Macleod I, Su AI. BioGPS and MyGene.info: organizing online, gene-centric information. *Nucleic Acids Res* 2013; **41**(D1): D561-5.
25. Hruz T, Laule O, Szabo G, et al. Genevestigator v3: a reference expression database for the meta-analysis of transcriptomes. *Adv Bioinformatics* 2008; **2008**: 420747.
26. Krupp M, Marquardt JU, Sahin U, Galle PR, Castle J, Teufel A. RNA-Seq Atlas--a reference database for gene expression profiling in normal tissue by next-generation sequencing. *Bioinformatics* 2012; **28**(8): 1184-5.
27. Uhlen M, Oksvold P, Fagerberg L, et al. Towards a knowledge-based Human Protein Atlas. *Nat Biotechnol* 2010; **28**(12): 1248-50.
28. Sievers F, Wilm A, Dineen D, et al. Fast, scalable generation of high-quality protein multiple sequence alignments using Clustal Omega. *Mol Syst Biol* 2011; **7**: 539.
29. Patton MA, Krywawych S, Winter RM, Brenton DP, Baraitser M. DOOR syndrome (deafness, onycho-osteodystrophy, and mental retardation): elevated plasma and urinary 2-oxoglutarate in three unrelated patients. *Am J Med Genet* 1987; **26**(1): 207-15.
30. van Bever Y, Balemans W, Duval EL, et al. Exclusion of OGDH and BMP4 as candidate genes in two siblings with autosomal recessive DOOR syndrome. *Am J Med Genet A* 2007; **143**(7): 763-7.
31. Kelley RI, Robinson D, Puffenberger EG, Strauss KA, Morton DH. Amish lethal microcephaly: a new metabolic disorder with severe congenital microcephaly and 2-ketoglutaric aciduria. *Am J Med Genet* 2002; **112**(4): 318-26.
32. Surendran S, Michals-Matalon K, Krywawych S, et al. DOOR syndrome: deficiency of E1 component of the 2-oxoglutarate dehydrogenase complex. *Am J Med Genet* 2002; **113**(4): 371-4.
33. James AW, Miranda SG, Culver K, Hall BD, Golabi M. DOOR syndrome: clinical report, literature review and discussion of natural history. *Am J Med Genet A* 2007; **143A**(23): 2821-31.
34. Rajab A, Riaz A, Paul G, Al-Khusaibi S, Chalmers R, Patton MA. Further delineation of the DOOR syndrome. *Clin Dysmorphol* 2000; **9**(4): 247-51.
35. Felix TM, de Menezes Karam S, Della Rosa VA, Moraes AM. DOOR syndrome: report of three additional cases. *Clin Dysmorphol* 2002; **11**(2): 133-8.
36. Uytterhoeven V, Kuenen S, Kasprowicz J, Miskiewicz K, Verstreken P. Loss of skywalker reveals synaptic endosomes as sorting stations for synaptic vesicle proteins. *Cell* 2011; **145**(1): 117-32.
37. Takeda K, Ishida A, Takahashi K, Ueda T. Synaptic vesicles are capable of synthesizing the VGLUT substrate glutamate from alpha-ketoglutarate for vesicular loading. *J Neurochem* 2012; **121**(2): 184-96.
